# A facile synthesis of functionalized 7,8-diaza[5]helicenes through an oxidative ring-closure of 1,1’-binaphthalene-2,2’-diamines (BINAMs)

**DOI:** 10.3762/bjoc.11.2

**Published:** 2015-01-05

**Authors:** Youhei Takeda, Masato Okazaki, Yoshiaki Maruoka, Satoshi Minakata

**Affiliations:** 1 Frontier Research Base for Global Young Researchers, Graduate School of Engineering, Osaka University, Yamadaoka 2-1, Suita, Osaka 565-0871, Japan; 2 Department of Applied Chemistry, Graduate School of Engineering, Osaka University, Yamadaoka 2-1, Suita, Osaka 565-0871, Japan

**Keywords:** aromatic amines, azahelicenes, cinnolines, halogen, oxidation

## Abstract

A facile and moderately functional-group-tolerant synthetic method for the preparation of 7,8-diaza[5]helicenes has been developed. It comprises of an oxidative ring-closing process of 1,1’-binaphthalene-2,2’-diamine (BINAM) derivatives with a chlorine-containing oxidant (*t*-BuOCl) in the presence of a base (2,6-lutidine). In addition the basic physicochemical properties of newly synthesized compounds have been investigated.

## Introduction

**Scheme 1:**
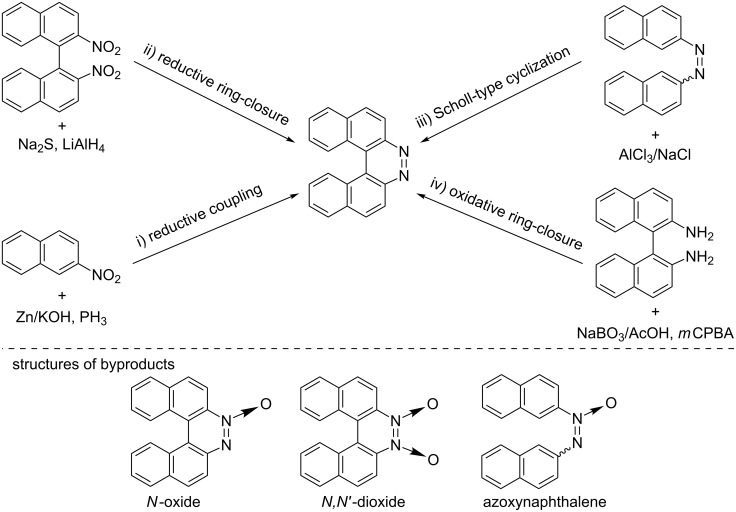
Synthetic routes to 7,8-diaza[5]helicene.

**Scheme 2:**
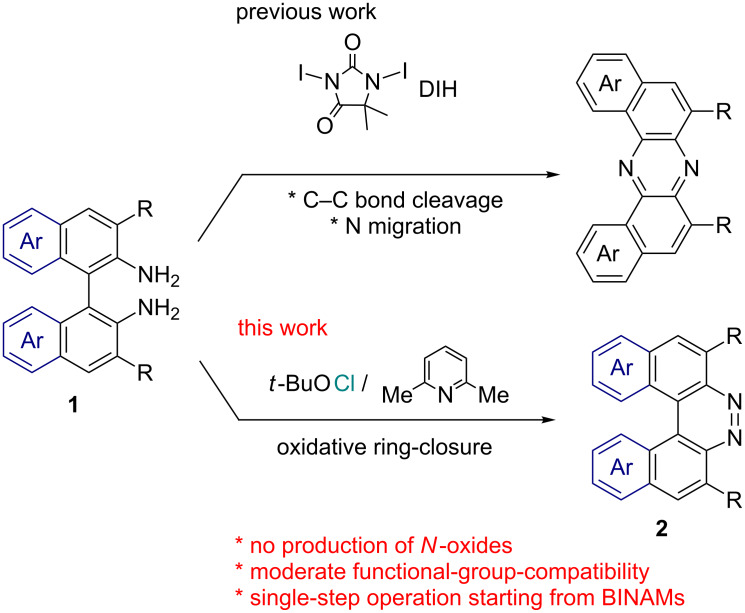
Oxidant-controlled transformations of BINAMs.

Helicenes, *ortho*-fused polycyclic aromatic compounds, have been fascinating organic chemists over the last century since the first synthesis of azahelicenes was reported by Meisenheimer in 1903 [1], not only because of their aesthetically attractive structures, but also because of their unique properties arising from the helical chirality [2–11]. In fact, helicenes have been finding more and more potential applications such as optoelectronic materials, asymmetric catalysts, and chemosensors [9]. Therefore, the development of synthetic methods for the preparation of helicenes which are difficult to access by conventional methodologies, would offer us many opportunities not only for creating helicene-based novel functional molecules but also uncovering missing pieces of unique aspects of helicenes. A bibliographic survey about helicenes led us focus on diazahelicenes having a diazene (–N=N–) moiety, which is a member of the azahelicene family [5]: These compounds would be a suitable model for probing the effects of the replacement of carbon atoms at the *K*-region of the carbohelicenes with nitrogen atoms on their structures and physicochemical properties [12–14] and should serve as ligands to metal complexes through the coordination at the diazene unit [15–17]. Taking 7,8-diaza[5]helicene as an example, conventional methodologies of preparing such compounds (Scheme 2) involve i) reductive coupling of 2-nitronaphthalenes using strong reductants like Zn dust [1,18] and PH_3_ [19], ii) reductive ring-closure of 2,2’-dinitro-1,1’-binaphthalenes using various reductants such as Na_2_S and LiAlH_4_ [20–21], and iii) the Scholl-type cyclization of 2,2’-azonaphthalenes using a eutectic melt of AlCl_3_/NaCl [22]. However, these methods require harsh reaction conditions such as the use of strong reductants or oxidants, leading to low functional group compatibility. More importantly, the reductive methods i) and ii) hardly avoid the production of *N*-oxide of diazahelicenes as well as azoxynaphthalenes (Scheme 1), the *N*-oxides are quantitatively reduced by a strong reductant to the corresponding diazahelicenes though. Another simple synthetic route to 7,8-diaza[5]helicenes would involve an oxidative ring-closure of 1,1’-binaphthalene-2,2’-diamines (BINAMs) (Scheme 1, iv). However, interestingly enough, this route remains to be explored. Corbett and Holt reported that the oxidation of BINAM with NaBO_3_ only gave a trace amount of the corresponding diazahelicene [23]. In this connection, Caronna and co-workers have recently reported a simple protocol for synthesizing 7,8-diaza[5]helicene through oxidation of BINAM with *m*CPBA and following reduction of its oxides [24]. Although the method is high yielding in 2 steps, it is not straightforward due to the formation of major amounts of its *N*-oxide and *N*,*N*’-dioxide (Scheme 1). Furthermore, functional group compatibility of the method still remains an open question, because of the involvement of the reduction process of the oxides employing LiAlH_4_. Therefore, in conjunction with recent advances in preparative methods of functionalized BINAMs [25–26], the development of oxidative ring-closing methods that allow for one-step and functional-group-tolerant synthesis of 7,8-diaza[5]helicenes from BINAMs would be desirable.

As a part of our project to develop oxidative transformations of aromatic amines for the construction of diverse aza-containing π-conjugated functional molecules [27–30], we have recently discovered an iodine-containing oxidant-induced unusual oxidative rearrangement of BINAMs leading exclusively to U-shaped azaacenes (Scheme 2, the upper reaction) [31]. During a thorough screening of oxidants for the rearrangement reaction, we found out that the use of *tert*-butyl hypochlorite (*t*-BuOCl) as an oxidant exclusively gave 7,8-diaza[5]helicene instead of the corresponding rearranged product in good yields. This preliminary finding prompted us to further explore the potential of this oxidative system as a facile and functional-group-tolerant synthetic method of this class of diazahelicenes, since no *N*-oxide byproducts are formed under this system and thereby any reduction processes using strong reductants are not needed. Herein we present a facile, straightforward, and moderately functional-group-tolerant synthesis of 7,8-diaza[5]helicenes (benzo[*f*]naphtho[2,1-*c*]cinnolines) bearing functional substituents on the helical π-conjugated backbone through an oxidative ring-closure of BINAM derivatives (Scheme 2, the lower reaction).

## Results and Discussion

Initially, we began to identify the optimum reaction conditions (oxidants, bases, and solvents) for the oxidative ring-closure of BINAMs using **1a** as the model substrate (Table 1, for the full details of the screening of reaction conditions, see Supporting Information File 1). As aforementioned in the Introduction section, we have recognized that the treatment of **1a** with 4 equivalents of chlorine-containing oxidant (*t*-BuOCl) exclusively gave diazahelicene **2a** in a high yield (Table 1, entry 1) [31]. As related works to this project, we have reported that the unique iodine-containing reagent *t*-BuOI, which is readily generated in situ from *t*-BuOCl and NaI, serves as a powerful oxidant for homo- and cross-dimerization of aromatic amines leading to aromatic azo compounds [27–28,30]. However, the employment of *t*-BuOI as an oxidant for this intramolecular oxidative N=N forming reaction resulted in much lower yield of **2a** (Table 1, entry 2) while the skeletal rearrangement product was obtained as a main product (51% yield) [31], showing the obvious superiority of *t*-BuOCl as an oxidant. Encouraged by these results, a variety of chlorine-containing oxidants were tested to probe the influence of oxidants on the chemical yields of **2a** (Table 1, entries 3–6). While the N-monochlorinated reagents *N*-chlorosuccinimide (NCS, entry 3) and *N*-chlorophthalimide (NCPh, entry 5) did not produce **2a** at all, di- and tri-chlorinated oxidants 1,3-dichloro-5,5-dimethylhydantoin (DCH, entry 4) and trichloroisocyanuric acid (TCCA, entry 6) gave low yields (ca. 20%) of **2a**. Solvents also significantly affected the yields of **2a** (Table 1, entries 7–11), and *t*-BuOH gave the best yield. In terms of the amounts of oxidant, the use of 2 equivalents of *t*-BuOCl (0.4 mmol) gave a lower yield of **2a** (54%, entry 12) with a moderate conversion of **1a**, in comparison with the case of using 4 equivalents of *t*-BuOCl (89%, entry 1). In light of the reaction stoichiometry of the oxidative process, 2 equivalents of HCl are supposed to be concomitantly generated, which could have protonated diamine **1a** leading to inhibition of conversion of **1a**. To trap the resulting HCl, the efficacy of base addition was investigated applying an ideal stoichiometry (2 equivalents) of *t*-BuOCl (Table 1, entries 13–17). The addition of an inorganic base (K_2_CO_3_) slightly improved the yield (Table 1, entry 13), while strong organic bases like DABCO (p*K*_a_ 8.93 in DMSO [32]) and DBU (p*K*_a_ 23.9 in MeCN [33]) gave a lower yield (32%) and no product, respectively (Table 1, entries 14 and 15). As results, the use of the moderately weak organic base 2,6-lutidine (p*K*_a_ 6.72 in water [34]) successfully afforded **2a** in high yield (Table 1, entry 16), and a small excess amount (2.2 equiv) of *t*-BuOCl and 2,6-lutidine gave diazahelicene **2a** quantitatively (Table 1, entry 17). When optically pure (*R*)-BINAM **1a** (99% ee) was applied to the optimized reaction conditions, racemic **2a** ([α]_D_^22^ = 0.0°, *c* 1.0, CHCl_3_) was obtained, losing the chiral information of **1a** through rapid racemization of optically active **2a** or of the reaction intermediates.

**Table 1:** The effect of reaction parameters on the oxidative ring-closure of **1a**.

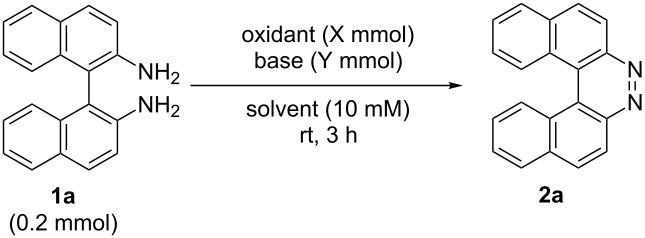					

Entry	Oxidant (X)	Base (Y)	Solvent	Yield (%)^a^	Recovery of **1a** (%)^a^

1	*t*-BuOCl (0.8)	—	*t*-BuOH	89^b^	0
2	*t*-BuOI (0.8)	—	*t*-BuOH	6^c^	0
3	NCS (0.8)	—	*t*-BuOH	0	63
4	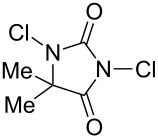 DCH (0.4)	—	*t*-BuOH	24^b^	0
5	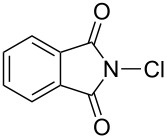 NCPh (0.8)	—	*t*-BuOH	0	95
6	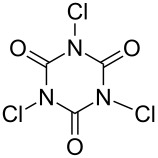 TCCA (0.27)	—	*t*-BuOH	20	0
7	*t*-BuOCl (0.8)	—	EtOH	61	0
8	*t*-BuOCl (0.8)	—	MeOH	58	0
9	*t*-BuOCl (0.8)	—	THF	30	0
10	*t*-BuOCl (0.8)	—	MeCN	22	0
11	*t*-BuOCl (0.8)	—	toluene	68	0
12	*t*-BuOCl (0.4)	—	*t*-BuOH	54	34
13	*t*-BuOCl (0.4)	K_2_CO_3_ (0.4)	*t*-BuOH	69	20
14	*t-*BuOCl (0.4)	DABCO (0.4)	*t*-BuOH	32	45
15	*t*-BuOCl (0.4)	DBU (0.4)	*t*-BuOH	0	34
16	*t*-BuOCl (0.4)	2,6-lutidine (0.4)	*t*-BuOH	90	10
**17**	***t*-BuOCl (0.44)**	**2,6-lutidine (0.44)**	***t-*BuOH**	**97^b^**	**0**

^a^Determined by ^1^H NMR. ^b^Isolated yields. ^c^Dibenzo[*a,j*]phenazine was obtained in 51% yield.

**Scheme 3:**
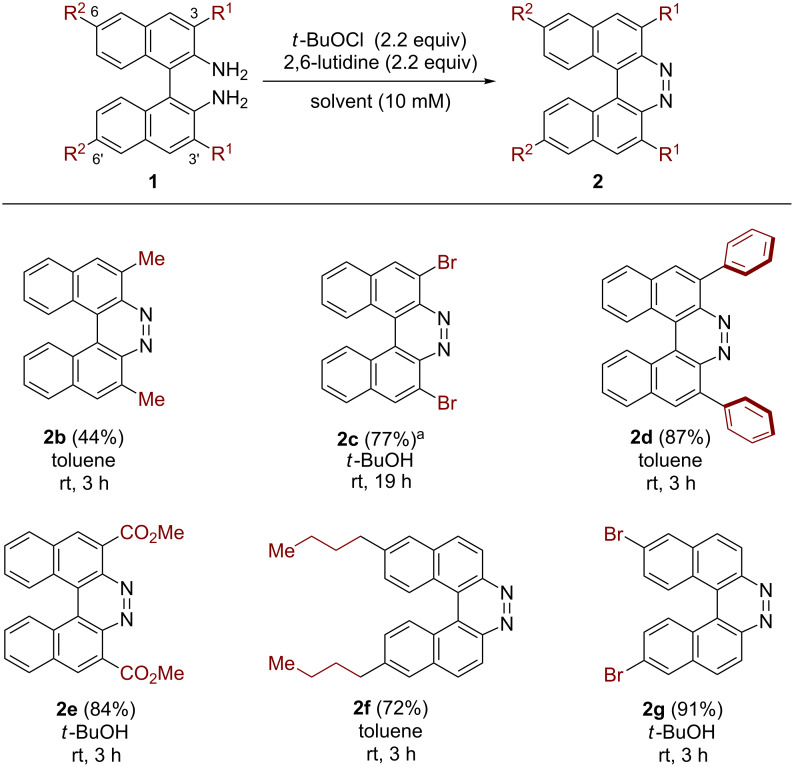
Substrate scope of the oxidative ring-closure of BINAMs. Reaction conditions: **1** (0.1 mmol), *t*-BuOCl (0.22 mmol), 2,6-lutidine (0.22 mmol), and solvent (10 mL) were stirred at the indicated temperature over the time shown below the respective product. Yields shown in parentheses indicate isolated yields. ^a^Run with 4 equivalents of *t*-BuOCl in the absence of 2,6-lutidine.

Having identified the optimized conditions for the oxidative ring-closure of **1a**, we then turned our attention to applying this method to the preparation of functionalized 7,8-diaza[5]helicenes (Scheme 3). When diamine **1b** bearing 3,3’-dimethyl substituents was subjected to the optimized conditions [*t*-BuOCl/2,6-lutidine (2.2 equiv) in *t*-BuOH at room temperature for 3 h], the desired helicene **2b** was obtained in a low yield (32%), even though electron-rich benzylic carbons labile to oxidative conditions are present. Modification studies (for the details, see the Supporting Information File 1) revealed that the use of toluene as a solvent slightly improved the yield of **2b** (Scheme 3). Notably, 3,3’-dibromo-substituted diamine **1c** gave a high yield of dibrominated diazahelicene **2c** even in the absence of 2,6-lutidine, albeit in a longer reaction time (Scheme 3). Such brominated helicene would be difficult to prepare by reductive methods using strong reductants like Zn dust, due to competitive over-reduction of the bromo functionality. It should be noted that the reaction efficiency of the oxidative process was not affected by the steric congestion around the aromatic amino moieties (Scheme 3, **2d**). Diazahelicene **2e** bearing two electron-withdrawing carboxylic ester groups was also successfully obtained in a high yield (84%) without impairing the ester functionality (Scheme 3). Again, it is worth emphasizing that such a type of diazahelicene would be difficult to prepare by conventional reductive methods. Furthermore, a BINAM derivative installed with two alkyl substituents at the 6,6’-position, **1f**, was efficiently transformed to the corresponding diazahelicene **2f** in a good yield. Bromo functionalities at the 6,6’-position of BINAM also survived the oxidative conditions to give the corresponding diazahelicene **2g** (Scheme 3). Regarding the limitation of this oxidative method, diamine substrates having strongly electron-donating substituents (both the 3,3’-MeO and 7,7’-MeO) were not successful, probably due to background side-reactions such as chlorination on the aromatic rings and oxidative oligomerization of BINAMs.

**Scheme 4:**
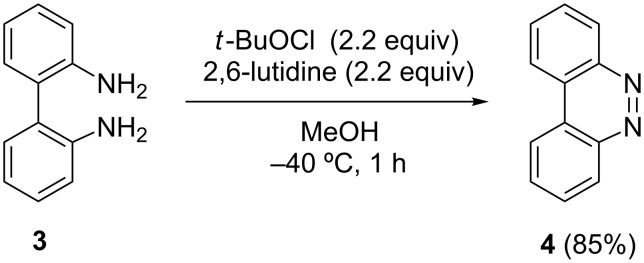
Oxidative ring-closure of **3**.

Furthermore, this new oxidative method was found to be applicable in the ring-closing reaction of 1,1’-biphenyl-2,2’-diamine (**3**) leading to benzo[*c*]cinnoline (**4**) in a good yield, although the modification of the reaction temperature was required (Scheme 4, for the detailed modification study of reaction conditions, see Supporting Information File 1).

**Scheme 5:**
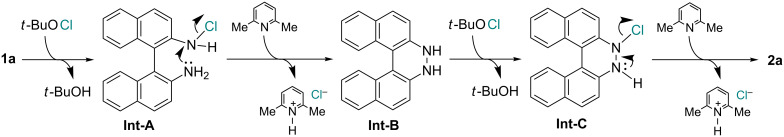
A possible reaction pathway of the oxidative ring-closure of **1a**.

A possible reaction pathway of the oxidative ring-closure of **1a** leading to **2a** is illustrated in Scheme 5. Chlorination of an amino group of **1a** with *t*-BuOCl would generate N-monochlorinated BINAM (**Int-A**) and release *t*-BuOH. The N-chlorination would induce umpolung reactivity of the amino moiety because of the attachment of electronegative halogen species on the N atom [27–30], and thereby an intramolecular nucleophilic unit (–NH_2_) would attack the electrophilic N-center to form N–N single bond (**Int-B**). The organic base (2,6-lutidine) would trap HCl generated through this process. Another repetition of a similar N-chlorination/HCl elimination cycle should give **2a** via **Int-C**.

**Figure 1:**
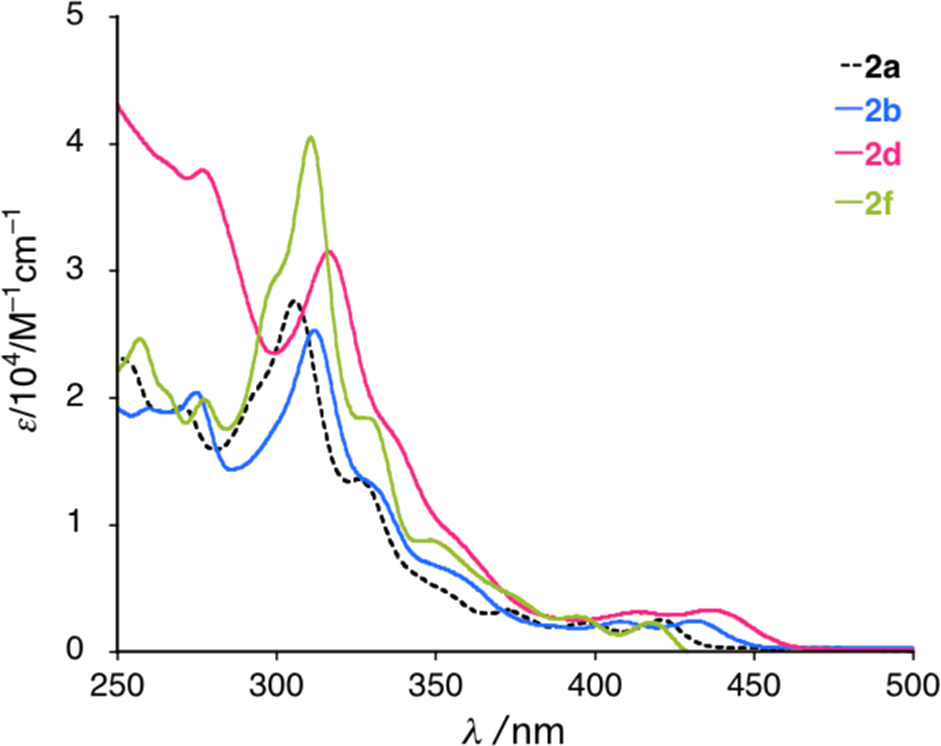
UV–vis absorption spectra of **2a**, **2b**, **2d**, and **2f**.

Since some of the synthesized diazahelicenes **2** are new members of 7,8-diaza[5]helicenes, basic physicochemical properties of **2a**–**2g** were also investigated (for the detailed data, see the Supporting Information File 1). Diluted dichloromethane solutions of **2** (ca. 10^−5^ M) exhibited UV–vis absorption spectra featuring weak absorptions ascribed to n–π* transitions in the lower energy region (400–450 nm), an absorption (shoulder at 330–350 nm) and a strong absorption (300–330 nm) ascribed to π–π* transitions, which are typical to aromatic ring-fused cinnoline derivatives [13] (representative spectra are shown in Figure 1; for the full spectra, see Supporting Information File 1). The introduction of two methyl (**2b**) and phenyl groups (**2d**) at the 3,3’-position of BINAM skeleton resulted in a red-shift of the absorption spectra over the whole region (Figure 1). In contrast, the introduction of 6,6’-*n*-Bu substituents (**2f**) caused a blue-shift of n–π* transitions (380–430 nm), while π–π* transitions (300–340 nm) were red-shifted. On the other hand, diluted dichloromethane solutions (10^−5^ M) of diazahelicenes **2** did not show photoluminescence (Φ_f_ < 0.02, for the detailed values of quantum yields, see the Supporting Information File 1).

Due to the presence of the electronegative diazene (–N=N–) moiety, diazahelicenes **2** are expected to possess stabilized LUMO energies. The LUMO energies, which were estimated from cyclic voltammetry (CV) experiments [*E*_LUMO_ = –(4.8 + ^red^*E*_1/2_) eV] using diluted CH_2_Cl_2_ solutions (10^−4^ M) of **2**, range from –2.92 to –3.13 eV (for the full cyclic voltammograms and the LUMO energies, see Supporting Information File 1). These LUMO energy levels fall in a similar range of the U-shaped azaacenes that our group previously reported [31] and well-known electron-transporting materials Alq_3_ [35], suggesting promising electron-accepting abilities of diazahelicenes.

## Conclusion

We have developed a facile and moderately functional-group-tolerant method for the synthesis of 7,8-diaza[5]helicenes through an efficient oxidative ring-closure of BINAMs with the combination of *t*-BuOCl as an oxidant and 2,6-lutidine as a base. This method has been applied to functionalized BINAMs except for highly electron-rich substrates to produce functionalized 7,8-diaza[5]helicenes in a single-step from BINAMs. Furthermore, basic properties of the azahelicenes have also been investigated.

## Supporting Information

File 1 Experimental procedures, characterization data, copies of NMR charts, UV–vis spectra, cyclic voltammograms, and TGA profiles.
