# Supplementary material for: A facile synthesis of functionalized 7,8-diaza[5]helicenes through an oxidative ring-closure of 1,1’-binaphthalene-2,2’-diamines (BINAMs)
Source: Beilstein J Org Chem. 2015 Jan 5;11:9–15. doi: 10.3762/bjoc.11.2 (PMC4311764; doi:10.3762/bjoc.11.2)
Supplement: File 1 — Experimental procedures, characterization data, copies of NMR charts, UV–vis spectra, cyclic voltammograms, and TGA profiles. [file Beilstein_J_Org_Chem-11-09-s001.pdf]

Supporting Information

for

A facile synthesis of functionalized 7,8-diaza[5]helicenes through an oxidative ring-closure of 1,1'-binaphthalene-2,2'-diamines (BINAMs)

Youhei Takeda*[1,2], Masato Okazaki[2], Yoshiaki Maruoka[2], and Satoshi Minakata*[2]

Address: [1]Frontier Research Base for Global Young Researchers, Graduate School of Engineering, Osaka University, Yamadaoka 2-1, Suita, Osaka 565-0871, Japan and [2]Department of Applied Chemistry, Graduate School of Engineering, Osaka University, Yamadaoka 2-1, Suita, Osaka 565-0871, Japan

Email: Youhei Takeda - takeda@chem.eng.osaka-u.ac.jp; Satoshi Minakata - minakata@chem.eng.osaka-u.ac.jp

*Corresponding author

Table of contents

General remarks	S2
Materials	S2–S3
Optimization studies of reaction conditions	S3–S6
Oxidative ring-closure of biaryldiamines **1** and **3**	S7–S11
Physicochemical properties	S12–S16
^{1}H and ^{13}C NMR spectra	S17–S23
References	S24

General remarks

All reactions were carried out under an atmosphere of nitrogen unless otherwise noted. Melting points were determined on a Stanford Research Systems MPA100 OptiMelt Automated Melting Point System. ^{1}H and ^{13}C NMR spectra were recorded on a JEOL JMTC-400/54/SS spectrometer (^{1}H NMR, 400 MHz; ^{13}C NMR, 100 MHz) using tetramethylsilane as an internal standard. Infrared spectra were acquired on a SHIMADZU IRAffinity-1 FT-IR Spectrometer. Mass spectra were obtained on a JEOL JMS-DX303HF mass spectrometer. High-resolution mass spectra were obtained on a JEOL JMS-DX303HF mass spectrometer. UV/vis spectra were recorded on a Shimadzu UV-2550 spectrophotometer. Emission spectra were recorded on a HAMAMATSU C11347-01 spectrometer with an integrating sphere. Cyclic voltammetry (CV) was performed with ALS-600 (BAS Inc.) system. Thermogravimetric analysis (TGA) was performed with TG/DTA-7200 (SII) system. Products were purified by chromatography on silica gel BW-300 and Chromatorex NH (Fuji Silysia Chemical Ltd.). Analytical thin-layer chromatography (TLC) was performed on pre-coated silica gel glass plates (Merck silica gel 60 F_{254} and Fuji Silysia Chromatorex NH, 0.25 mm thickness). Compounds were visualized with UV lamp. Optical rotations were measured in a thermostated conventional 10 cm cell on a JASCO P-2200 polarimeter using the sodium-D line (589 nm).

Materials

1,1'-Binaphthalene-2,2'-diamine (BINAM) was purchased from Sigma-Aldrich and used as received. DBU, triethylamine, and 2,6-lutidine were distilled with Kugelrohr apparatus, and other commercial reagents were purchased from Sigma-Aldrich, TCI, or Wako Pure Chemical Industries, Ltd. and used as received. Alcohol solvents were dried over activated molecular sieves 3A. THF, CH_3CN, and Et_2O were purchased as dehydrated grade and dried by passing through a glass contour solvent dispensing system (Nikko Hansen & Co., Ltd.). Dehydrated CH_2Cl_2 and toluene were purchased from Kanto Chemical Co., Inc. and used as received. DMF was distilled using $CaSO_4$ as a dehydrating agent. CH_2Cl_2 (fluorescence spectroscopic grade) was purchased from Kanto Chemical Co., Inc. for the measurement of UV-vis and emission spectra. Biaryldiamines **1b**[S1] [360779-01-7], **1c**[S2] [1051425-55-8, (*R*)-enantiomer], **1d**[S3] [1229013-43-7], **1e**[S4] [155855-47-3], **1g**[S5] [861890-12-2], and **3**[S6] [1454-80-4] were prepared according to the procedures in literature.

Preparation of 6,6'-di-*n*-butyl-1,1'-binaphthalene-2,2'-diamine (1f)

Biaryldiamine **1f** was prepared by modified cross-coupling method[S7] from biaryldiamine **1g** as follows (Scheme S1): THF was degassed through freeze-pump-thaw cycling for three times before used. To a round-bottomed flask (50 mL) equipped with a

magnetic stir bar, were added biaryldiamine **1g** (1.326 g, 3.0 mmol), $Pd(OAc)_2$ (13.4 mg, 0.06 mmol), and SPhos (49.2 mg, 0.12 mmol) under the air. The tube was capped with a rubber septum, evacuated, and then refilled with N_2 gas for three times. THF (2 mL) and 0.5 M THF solution of *n*-BuZnBr (14.4 mL, 7.2 mmol) were added to the tube through the septum, and the mixture was stirred under N_2 atmosphere at room temperature for 12 h. To the reaction mixture, was added saturated aqueous NH_4Cl solution (10 mL), and the resulting mixture was extracted with EtOAc (20 mL × 3). The organic extract was dried over Na_2SO_4 and concentrated under vacuum to give the crude product. Purification by flash column chromatography on silica gel (eluent: hexane/EtOAc 8:2) gave biaryldiamine **1f** as pale brown solid (1.051 g, 88%). mp 130 °C (dec.); R_f 0.13 (hexane/EtOAc 8:2); ^{1}H NMR (400 MHz, $CDCl_3$) δ 0.92 (t, J = 7.2 Hz, 6H), 1.37 (tq, J = 7.2, 7.6 Hz, 4H), 1.63 (tt, J = 7.6, 7.6 Hz, 4H), 2.68 (t, J = 7.6 Hz, 4H), 3.60 (br, 4 H), 7.01 (d, J = 8.8 Hz, 2H), 7.05 (dd, J = 1.6, 8.8 Hz, 2H), 7.11 (d, J = 8.8 Hz, 2H), 7.56 (d, J = 1.6 Hz, 2H), 7.72 (d, J = 8.8 Hz, 2H); ^{13}C NMR (100 MHz, $CDCl_3$) δ 13.9, 22.4, 33.5, 35.4, 112.9, 118.3, 123.9, 126.6, 128.3, 128.6, 128.8, 132.0, 136.8, 141.9; IR (ATR) ν 2929, 1606, 1500, 1382, 1282, 824, 810 cm^{-1}; MS (EI): *m/z* (relative intensity, %) 396 (M^+, 100), 353 ($[C_{25}H_{25}N_2]^+$, 45); HRMS (EI): *m/z* calcd for $C_{28}H_{32}N_2$ (M) 396.2565, found 396.2568.

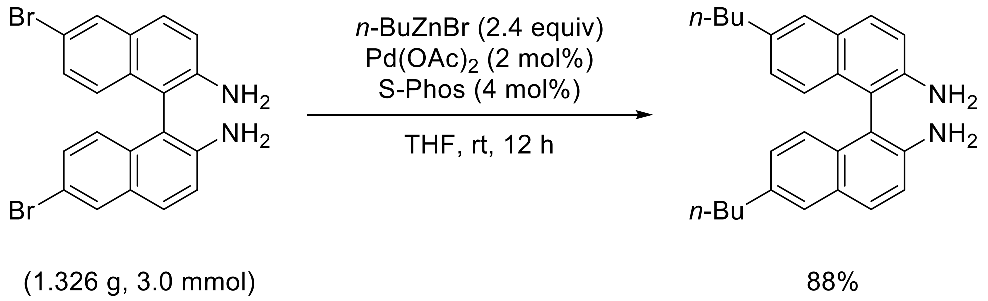

Scheme S1. Preparation of **1f**.

Optimization studies of reaction conditions

A typical procedure for the optimization studies using 1a as substrate

To a two-necked reaction tube (20 mL, entry 2 in Table S1) or a two-necked round-bottomed flask (50 mL, entries 1 and 3–11 in Table S1, Table S2, and Table S3) equipped with a magnetic stir bar, was added 1,1'-binaphthalene-2,2'-diamine (**1a**) (0.2 mmol) under the air. The vessel was capped with a rubber septum and evacuated and refilled with N_2 gas for three times, and an appropriate solvent was added through the septum. To the mixture, were added an additive and an appropriate oxidant under a stream of N_2 gas at the indicated temperature. The resulting solution was stirred for indicated time before quenched with aqueous $Na_2S_2O_3$ solution (1.0 M, 20 mL), and the resulting mixture was extracted with

CH_2Cl_2 (20 mL × 3). The combined organic extracts were dried over Na_2SO_4 and concentrated under vacuum to give the crude product. The yields of products were calculated by the integration of ^{1}H NMR signals of the crude product. Separation by flash column chromatography on silica gel gave product **2a**.

7,8-Diaza[5]helicene (2a) [188-55-6]

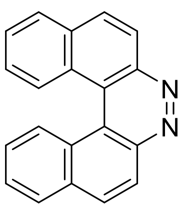

Spectroscopic data were in good agreement with those previously reported.[S8] The spectroscopic data are also available in our previous paper.[S9] Purified by flash column chromatography on silica gel (eluent: hexane/EtOAc, 99:1 to 5:5) and recrystallization from $CHCl_3$; Yellow solid; R_f 0.15 (hexane/EtOAc 8:2); MS (EI): *m/z* (relative intensity, %) 280 (M^+, 70), 252 ($[C_{20}H_{12}]^+$, 100); HRMS (EI): *m/z* calcd for $C_{20}H_{12}N_2$ (M) 280.1000, found 280.1002.

Table S1. The effect of solvents.

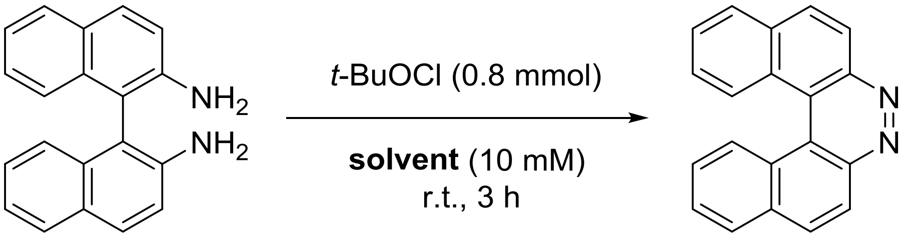

1a (0.2 mmol) **2a**

entry	solvent	yield (%)[a]	recovery of **1a** (%)[a]
1	*t*-BuOH	89[b]	0
2	*t*-BuOH[c]	54	0
3	THF	30	0
4	CH_3CN	22	0
5	CH_2Cl_2	52	0
6	toluene	68	0
7	DMF	62	0
8	Et_2O	37	0
9	2-propanol	47	48
10	EtOH	61	0
11	MeOH	58	0

[a] ^{1}H NMR yields. [b] isolated yield. [c] 40 mM.

Table S2. The effect of chlorine-containing oxidants.

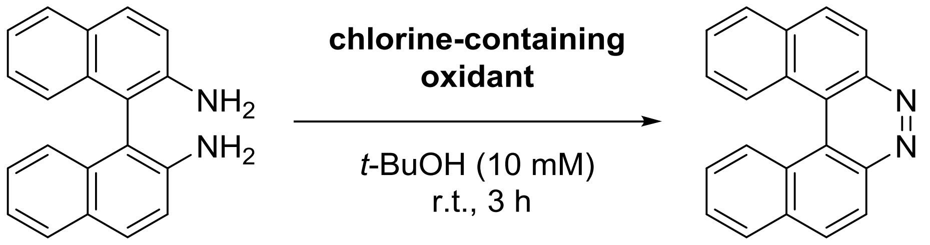

1a (0.2 mmol) **2a**

entry	**chlorine-containing oxidant** (mmol)	yield (%)[a]	recovery of **1a** (%)[a]
1	NCS (0.8)	0	63
2	DCH (0.4)	24[b]	0
3	DCH (0.8)	57	0
4	NCPh (0.8)	0	95
5	TCCA (0.27)	20	0

[a] ^{1}H NMR yields. [b] isolated yield.

Structures of chlorine-containing oxidants:

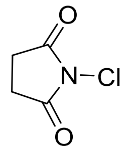

NCS
N-chlorosuccinimide

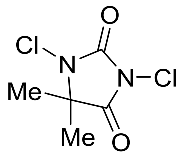

DCH
1,3-dichloro-5,5-dimethylhydantoin

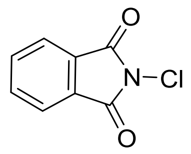

NCPh
N-chlorophthalimide

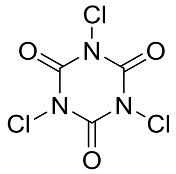

TCCA
trichloroisocyanuric acid

Table S3. The effect of equivalents of *t*-BuOCl and bases.

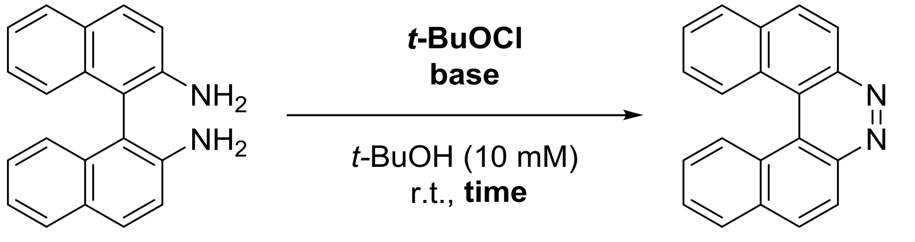

1a (0.2 mmol) **2a**

entry	***t*-BuOCl** (mmol)	**base** (mmol)	**time** (h)	yield (%)[a]	recovery of **1a** (%)[a]
1	0.6	-	3	93	0
2	0.4	-	3	54	34
3	0.4	-	24	71	19
4	0.4	K_2CO_3 (0.4)	3	69	20
5	0.4	DABCO (0.4)	3	32	45
6	0.4	DBU (0.4)	3	0	34
7	0.4	NEt_3 (0.4)	3	0	65
8	0.4	pyridine (0.4)	3	33	40
9	0.4	2,6-di-*tert*-butylpyridine (0.4)	3	77	19
10	0.4	2,6-lutidine (0.4)	3	90	10
11	0.4	2,6-lutidine (0.4)	7	92	8
12	0.44	2,6-lutidine (0.44)	3	97[b]	0

[a] ^{1}H NMR yields. [b] isolated yield.

Structures of bases

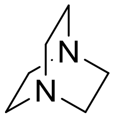

DABCO
1,4-diazabicyclo[2.2.2]octane

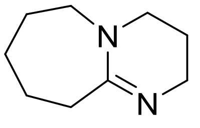

DBU
1,8-diazabicyclo[5.4.0]undec-7-ene

Oxidative ring-closure of biaryldiamines 1 and 3

A typical procedure for the oxidative ring-closure of biaryldiamines 1 and 3

To a two-necked round-bottomed flask (50 mL) equipped with a magnetic stir bar, was added biaryldiamine **1** (or **3**) (0.1 mmol) under the air. The flask was capped with a

rubber septum, evacuated, and refilled with N_2 gas for three times. Solvent (10 mL) and 2,6-lutidine (23.5 mg, 0.22 mmol or none) were added to the tube through the septum. To the mixture, was added *t*-BuOCl (23.8 mg, 0.22 mmol or 43.4 mg, 0.40 mmol) through the septum at the indicated temperature. The resulting solution was stirred for the indicated time (Table 2 in the text) before quenched with aqueous $Na_2S_2O_3$ solution (1.0 M, 20 mL), and the resulting mixture was extracted with CH_2Cl_2 (20 mL × 3). The combined organic extracts were dried over Na_2SO_4 and concentrated under vacuum to give the crude product. Purification by flash column chromatography on silica gel gave the corresponding 7,8-diaza[5]helicene (for example, compound **2a**: 27.2 mg, 97%).

Table S4. The oxidative ring-closure of biaryldiamine **1b**.

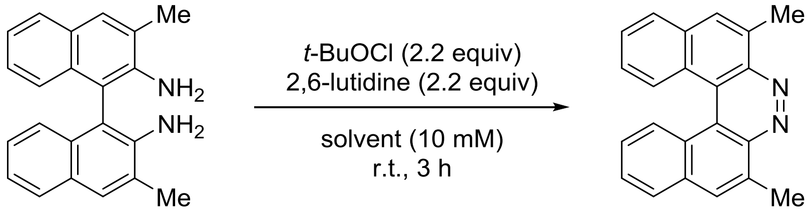

1b
(31.2 mg, 0.1 mmol)

2b

entry	solvent	yield (%)[a]	recovery of **1b** (%)[a]
1	*t*-BuOH	32	22
2	MeOH	17	28
3	CH_2Cl_2	16	trace
4	toluene	44[b]	11[b]

[a] ^{1}H NMR yields. [b] isolated yield.

6,9-Dimethyl-7,8-diaza[5]helicene (2b)

Purified by flash column chromatography on NH silica gel (eluent: hexane/EtOAc 99:1) and recrystallization from hexane; Yellow solid (13.6 mg, 44%); mp 196 °C (dec.); R_f 0.33 (hexane/EtOAc 8:2, NH); ^{1}H NMR (400 MHz, $CDCl_3$) δ 3.16 (s, 6H), 7.31 (dd, J = 8.0, 8.0 Hz, 2H), 7.64 (dd, J = 8.0, 8.0 Hz, 2H), 7.91 (d, J = 8.0 Hz, 2H) , 7.95 (s, 2H), 8.71 (d, J = 8.0 Hz, 2H); ^{13}C NMR (100 MHz, $CDCl_3$) δ 18.5, 120.0, 124.5, 127.3, 127.5, 129.2, 129.5, 129.8, 134.0, 134.2, 145.9; IR (ATR) ν 2922, 1734, 1457, 1425, 1261, 1160, 1116, 889, 753 cm^{-1}; MS (EI): *m/z* (relative intensity, %) 308 (M^+, 100), 280 ($[C_{22}H_{16}]^+$, 31); HRMS (EI): *m/z* calcd for $C_{22}H_{16}N_2$ (M) 308.1313, found 308.1312.

Table S5. The oxidative ring-closure of biaryldiamine **1c**

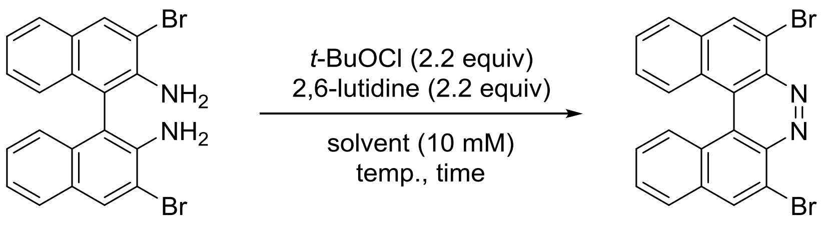

1c
(44.2 mg, 0.1 mmol)

2c

entry	solvent	temp. (°C)	time (h)	yield (%)[a]	recovery of **1c** (%)[a]
1	*t*-BuOH	rt	20	44[b]	15[b]
2[c]	*t*-BuOH	rt	19	77[b]	0
3	*t*-BuOH	60	24	36	12
4	MeOH	rt	9	9	28
5	toluene	rt	24	15	0

[a] ^{1}H NMR yields. [b] isolated yield. [c] *t*-BuOCl (4.0 equiv), 2,6-lutidine (0 equiv)

6,9-Dibromo-7,8-diaza[5]helicene (2c)

Parts of spectroscopic data are available in a literature.[S10] Purified by flash column chromatography on silica gel (eluent: hexane/EtOAc 99:1–95:5) and recrystallization from hexane/$CHCl_3$; Yellow solid (19.3 mg, 44% or 33.7 mg, 77%); mp 307 °C (dec.); R_f 0.40 (hexane/EtOAc 8:2); ^{1}H NMR (400 MHz, $CDCl_3$) δ 7.41 (dd, J = 8.0, 8.0 Hz, 2H), 7.71 (dd, J = 8.0, 8.0 Hz, 2H), 7.94 (d, J = 8.0 Hz, 2H), 8.51 (s, 2H), 8.64 (d, J = 8.0 Hz, 2H); ^{13}C NMR (100 MHz, $CDCl_3$) δ 121.3, 121.6, 126.0, 127.4, 127.5, 129.5, 130.3, 134.4, 134.5, 143.6; IR (ATR) ν 3053, 1591, 1383, 1250, 1108, 960, 890, 837, 773, 753 cm^{-1}; MS (EI): *m/z* (relative intensity, %) 438 (M^+, 50), 357 ($[C_{20}H_{10}BrN_2]^+$, 14), 278 ($[C_{20}H_{10}N_2]^+$, 23), 250 ($[C_{20}H_{10}]^+$, 100); HRMS (EI): *m/z* calcd for $C_{20}H_{10}Br_2N_2$ (M) 435.9211, found 435.9210.

Table S6. The oxidative ring-closure of biaryldiamine **1d**.

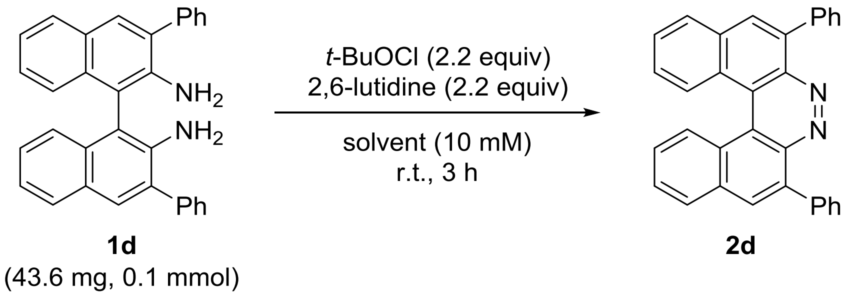

entry	solvent	yield (%)^a	recovery of **1d** (%)^a
1	*t*-BuOH	33^b	18^b
2	MeOH	28	24
3	toluene	87^b	0

^{a} ^{1}H NMR yields. ^{b} isolated yield.

6,9-Diphenyl-7,8-diaza[5]helicene (2d)

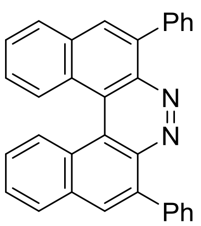

Purified by flash column chromatography on silica gel (eluent: hexane/EtOAc 97:3) and recrystallization from hexane/$CHCl_3$; Yellow solid (37.6 mg, 87%); mp 239 °C (dec.); R_f 0.35 (hexane/EtOAc 8:2); ^{1}H NMR (400 MHz, $CDCl_3$) δ 7.43–7.45 (m, 4H), 7.50 (dd, J = 7.6, 7.6 Hz, 4H), 7.72 (dd, J = 7.6, 7.6 Hz, 2H), 7.82 (d, J = 6.8 Hz, 4H), 8.06 (d, J = 7.6 Hz, 2H), 8.17 (s, 2H), 8.79 (d, J = 8.8 Hz, 2H); ^{13}C NMR (100 MHz, $CDCl_3$) δ 120.1, 125.4, 127.7, 128.0, 128.1, 128.3, 129.4, 129.5, 130.9, 131.2, 133.8, 137.8, 138.4, 144.6; IR (ATR) ν 3027, 1494, 1445, 898, 766, 754 cm^{-1}; MS (EI): *m/z* (relative intensity, %) 432 (M^+, 67), 431 ($[C_{32}H_{19}N_2]^+$, 100); HRMS (EI): *m/z* calcd for $C_{32}H_{20}N_2$ (M) 432.1626, found 432.1624.

Dimethyl 7,8-diaza[5]helicene-6,9-dicarboxylate (2e)

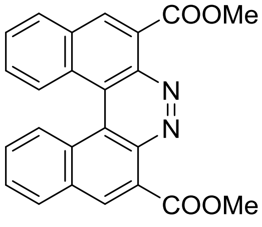

Purified by flash column chromatography on silica gel (eluent: hexane/EtOAc 8:2–5:5) and recrystallization from hexane/$CHCl_3$; Yellow solid (33.3 mg, 84%); mp 215 °C (dec.); R_f 0.35 (hexane/EtOAc 5:5); ^{1}H NMR (400 MHz, $CDCl_3$) δ 4.14 (s, 6H), 7.48 (dd, J = 8.0, 8.0 Hz, 2H), 7.75 (dd, J = 8.0, 8.0 Hz, 2H), 8.07 (d, J = 8.0 Hz, 2H), 8.45 (s, 2H), 8.69 (d, J = 8.0 Hz, 2H); ^{13}C NMR (100 MHz, $CDCl_3$) δ 53.1, 119.7,

127.2, 128.8, 129.1, 129.2, 129.4, 130.1, 132.1, 132.6, 143.3, 168.0; IR (ATR) ν 2942, 1724, 1425, 1269, 1220, 1137, 781, 761 cm^{-1}; MS (EI): *m/z* (relative intensity, %) 396 (M^+, 32), 338 ($[C_{22}H_{14}N_2O_2]^+$, 100), 250 ($[C_{20}H_{10}]^+$, 18); HRMS (EI): *m/z* calcd for $C_{24}H_{16}N_2O_4$ (M) 396.1110, found 396.1112.

Table S7. The oxidative ring-closure of biaryldiamine **1f**.

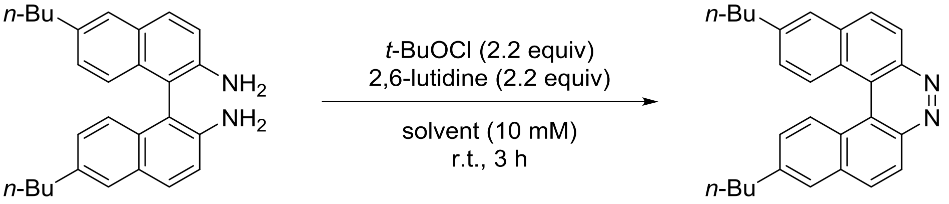

1f
(39.6 mg, 0.1 mmol)

2f

entry	solvent	yield (%)[a]	recovery of **1f** (%)[a]
1	*t*-BuOH	49	23
2	MeOH	26	0
3	toluene	72[b]	0

[a] ^{1}H NMR yields. [b] isolated yield.

3,12-Di-*n*-butyl-7,8-diaza[5]helicene (2f)

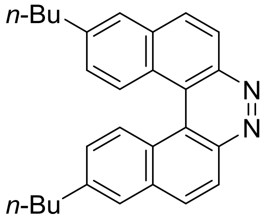

Purified by flash column chromatography on silica gel (eluent: hexane/EtOAc 9:1–8:2) and recrystallization from hexane; Pale yellow solid (28.2 mg, 72%); mp 161 °C (dec.); R_f 0.18 (hexane/EtOAc 8:2); ^{1}H NMR (400 MHz, $CDCl_3$) δ 0.99 (t, J = 7.6 Hz, 6H), 1.44 (tq, J = 7.6, 7.6 Hz, 4H), 1.76 (tt, J = 7.6, 7.6 Hz, 4H), 2.86 (t, J = 7.6 Hz, 4H), 7.28 (dd, J = 1.6, 8.4 Hz, 2H), 7.80 (d, J = 1.6 Hz, 2H), 8.09 (d, J = 8.8 Hz, 2H), 8.49 (d, J = 8.8 Hz, 2H), 8.75 (d, J = 8.4 Hz, 2H); ^{13}C NMR (100 MHz, $CDCl_3$) δ 13.9, 22.5, 33.3, 35.7, 120.0, 126.1, 126.5, 126.9, 129.1, 130.5, 134.4, 144.6, 146.6 (one carbon is unsatisfied, probably due to the overlap of signals.); IR (ATR) ν 2930, 1618, 1464, 1259, 1105, 891, 827, 810 cm^{-1}; MS (EI): *m/z* (relative intensity, %) 392 (M^+, 100), 364 ($[C_{28}H_{28}]^+$, 15), 349 ($[C_{27}H_{25}]^+$, 12), 335 ($[C_{26}H_{23}]^+$, 31), 321 ($[C_{25}H_{21}]^+$, 17), 307 ($[C_{24}H_{19}]^+$, 47); HRMS (EI): *m/z* calcd for $C_{28}H_{28}N_2$ (M) 392.2252, found 392.2251.

3,12-Dibromo-7,8-diaza[5]helicene (2g)

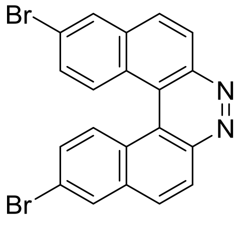

Purified by flash column chromatography on silica gel (eluent: hexane/EtOAc 9:1–7:3) and recrystallization from $CHCl_3$; Brown solid (39.9 mg, 91%); mp 205 °C (dec.); R_f 0.18 (hexane/EtOAc 8:2); ^1H NMR (400 MHz, $CDCl_3$) δ 7.55 (dd, J = 2.0, 8.8 Hz, 2H), 8.09 (d, J = 8.8 Hz, 2H), 8.20 (d, J = 2.0 Hz, 2H), 8.58 (d, J = 8.8 Hz, 2H), 8.61 (d, J = 8.8 Hz, 2H); ^{13}C NMR (100 MHz, $CDCl_3$) δ 119.4, 123.8, 126.4, 127.9, 129.0, 129.8, 130.4, 130.5, 135.6, 146.5; IR (ATR) ν 3061, 1591, 1495, 1436, 1263, 1086, 876, 852, 833, 820, 803 cm^{-1}; MS (EI): m/z (relative intensity, %) 438 (M^+, 69), 278 ($[C_{20}H_{10}N_2]^+$, 79), 250 ($[C_{20}H_{10}]^+$, 100); HRMS (EI): m/z calcd for $C_{20}H_{10}N_2Br_2$ (M) 435.9211, found 435.9208.

Table S8. The oxidative ring-closure of biaryldiamine **3**.

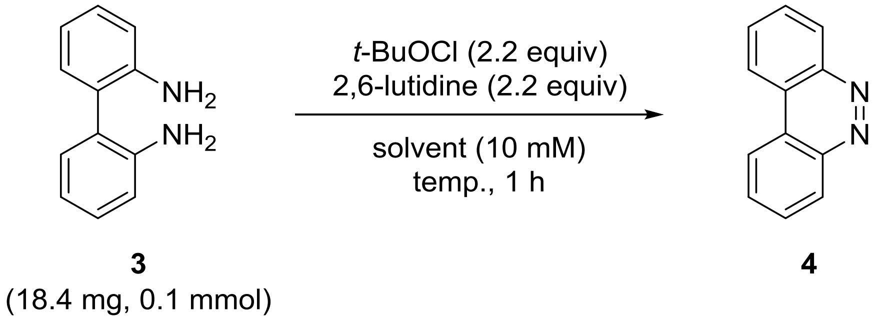

entry	solvent	temp. (°C)	yield (%)[a]	recovery of **3** (%)[a]
1	*t*-BuOH	rt	53	trace
2	MeOH	rt	42	0
3	MeOH	0	65[b]	0
4	MeOH	-40	85[b]	0

[a] ^1H NMR yields. [b] isolated yields.

Benzo[*c*]cinnoline (4) [230-17-1]

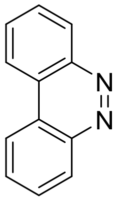

Spectroscopic data were in agreement with those previously reported.[S11] The spectroscopic data are also available in our previous paper.[S9] Purified by flash column chromatography on silica gel (hexane/EtOAc 95:5 to 8:2); Yellow solid; R_f 0.18 (hexane/EtOAc 8:2).

Physicochemical properties

Table S9. Summary of physicochemical properties of diazahelicenes **2** and cinnoline **4**.

	Absorption		Emission (solution)		CV (vs. Fc/Fc$^+$)				TGA
	λ_{max} (nm)	ε ($M^{-1}cm^{-1}$)	λ_{ex} (nm)	Φ_{FL}	$^{red}E_{pc}$ (V)	$^{red}E_{pa}$ (V)	$^{red}E_{1/2}$ (V)	LUMO level (eV)	T_d (5wt% loss) (°C)
**2a**	306, 398, 421	27672, 2294, 2509	300	0.02	−1.92	−1.83	−1.88	−2.92	251
**2b**	312, 408, 431	25282, 2403, 2403	300	<0.01	-	-	-	-	268
**2c**	318, 410, 434	30326, 2580, 2688	300	<0.01	−1.70	−1.62	−1.66	−3.13	306
**2d**	316, 413, 436	31470, 3157, 3266	300	<0.01	−1.93	−1.80	−1.86	−2.93	222
**2e**	309, 397, 419	33950, 3223, 3223	300	<0.01	−1.74	−1.61	−1.67	−3.12	315
**2f**	311, 392, 418	40513, 2715, 2280	300	<0.01	-	-	-	-	300
**2g**	310, 395, 418, 445	31106, 3635, 4342, 2524	300	<0.01	−1.80	−1.72	−1.76	−3.03	277
**4**	298,308,351, 357	8990,9192, 1515, 1313	300	0.15	-	-	-	-	172

UV–vis and emission spectra

CH_2Cl_2 (fluorescence spectroscopic grade) was purged with N_2 for 30 min before the measurements. UV-vis and emission spectra of diazahelicenes **2** and cinnoline **4** were measured at room temperature using CH_2Cl_2 solutions (1.0×10^{-5} M).

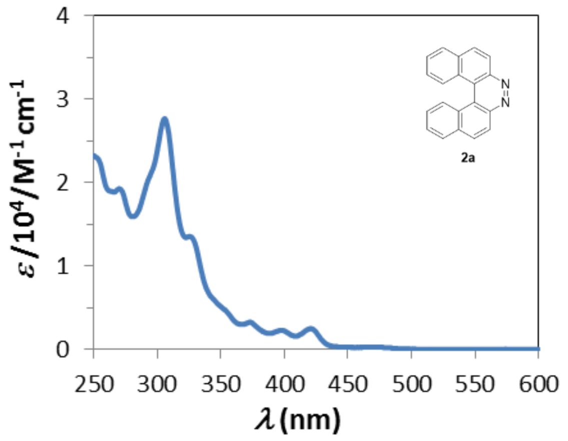

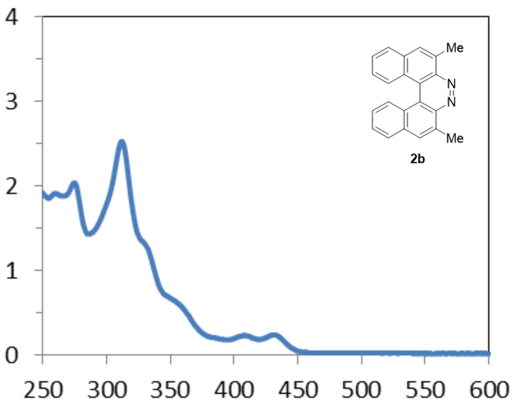

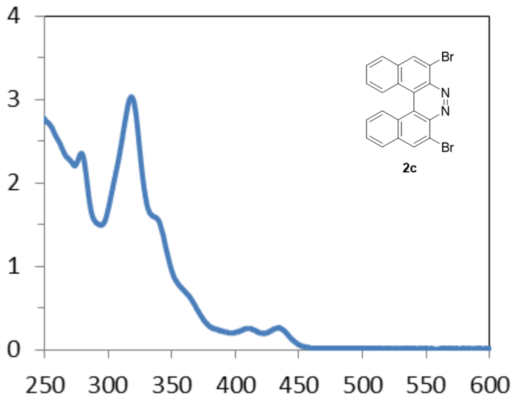

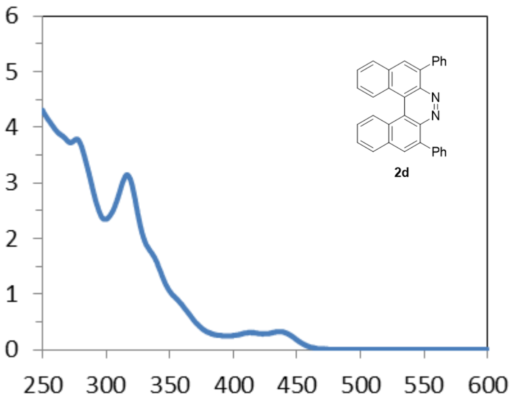

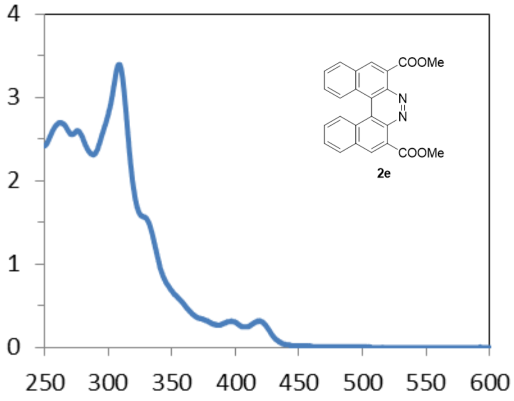

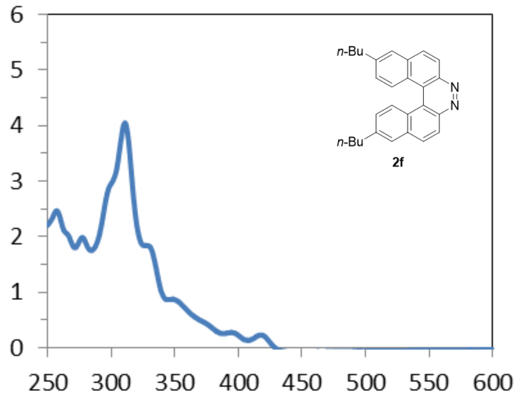

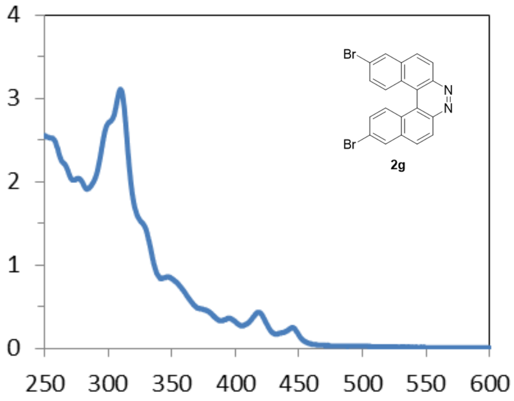

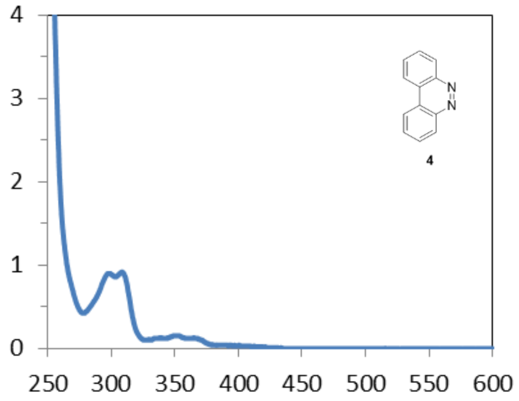

Cyclic voltammetry

Cyclic voltammetry experiments were conducted at room temperature with CH_2Cl_2 solutions of diazahelicenes **2** and cinnoline **4** (5.0×10^{-4} M) containing 0.1 M tetrabutylammonium hexafluorophosphate as a supporting electrolyte in a cell equipped with a Pt as the working electrode (scanning rate: 100 m/V). A Pt wire and an Ag wire were applied as the counter and the reference electrode, respectively. All the potentials were corrected against the Fc/Fc^+ (Fc = ferrocene) couple and the values of LUMO levels were calculated with the equation S1.

$$\text{LUMO} = -(4.8 + {}^{red}E_{1/2} \text{ vs. } Fc/Fc^{+}) \qquad \text{(S1)}$$

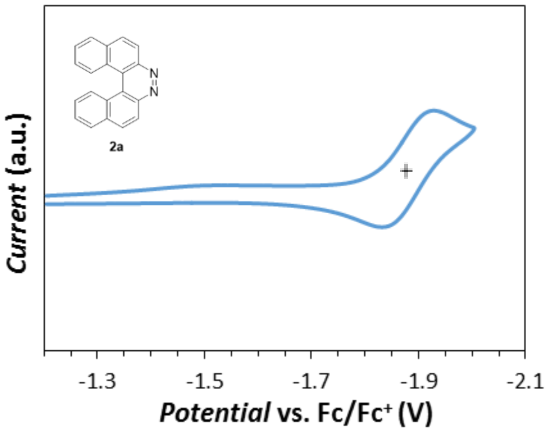
2a
N
N
Current (a.u.)
-1.3
-1.5
-1.7
-1.9
-2.1
Potential vs. Fc/Fc+ (V)

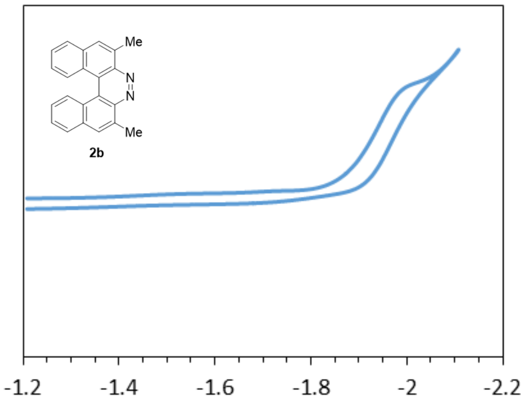
Me
N
N
Me
2b
-1.2
-1.4
-1.6
-1.8
-2
-2.2

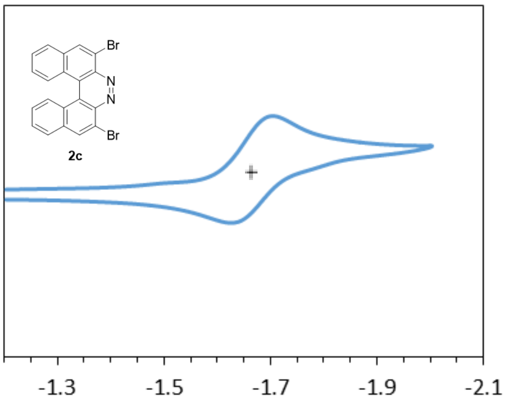
Br
N
N
Br
2c
-1.3
-1.5
-1.7
-1.9
-2.1

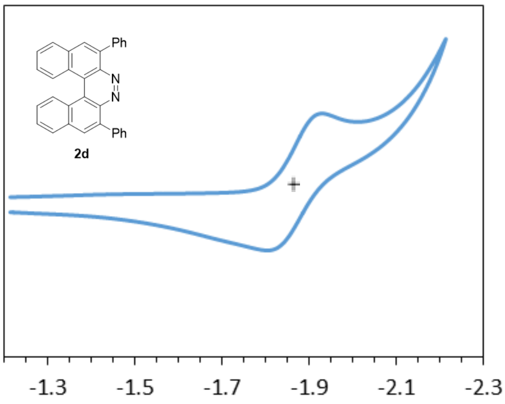
Ph
N
N
Ph
2d
-1.3
-1.5
-1.7
-1.9
-2.1
-2.3

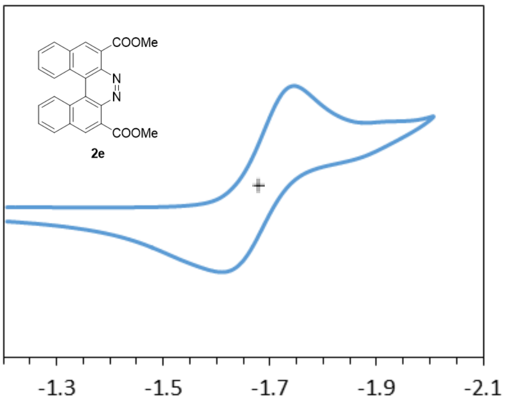
COOMe
N
N
COOMe
2e
-1.3
-1.5
-1.7
-1.9
-2.1

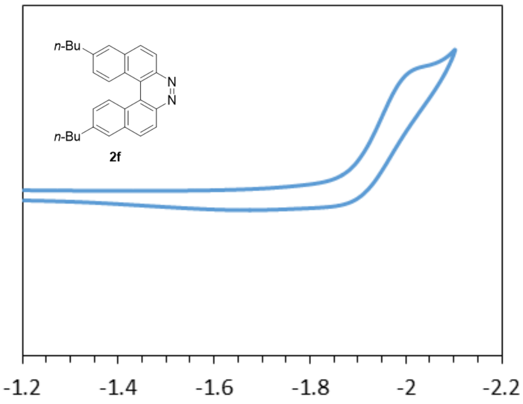
n-Bu
N
N
n-Bu
2f
-1.2
-1.4
-1.6
-1.8
-2
-2.2

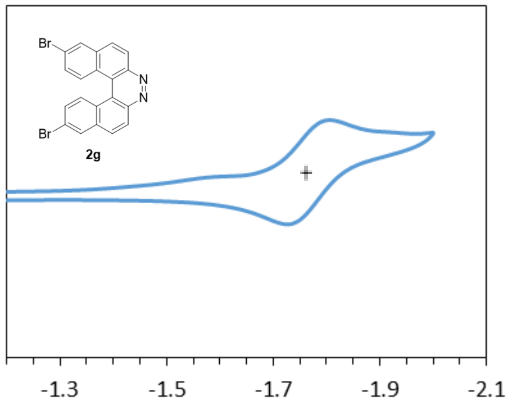

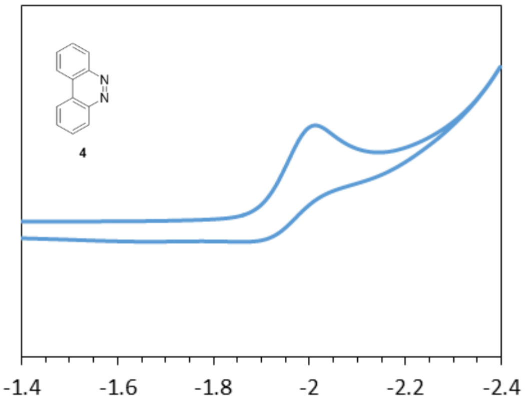

Thermogravimetric analysis (TGA)

All the TGA profiles of diazahelicenes **2** and cinnoline **4** were measured under the nitrogen flow (200 mL/min), starting from 40 °C to 600 °C at the ramp rate of 10 °C/min.

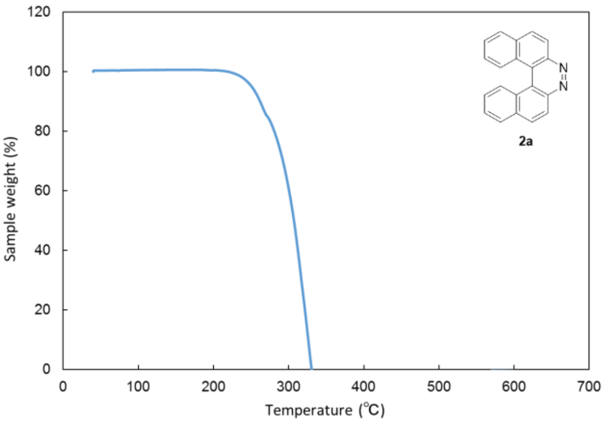

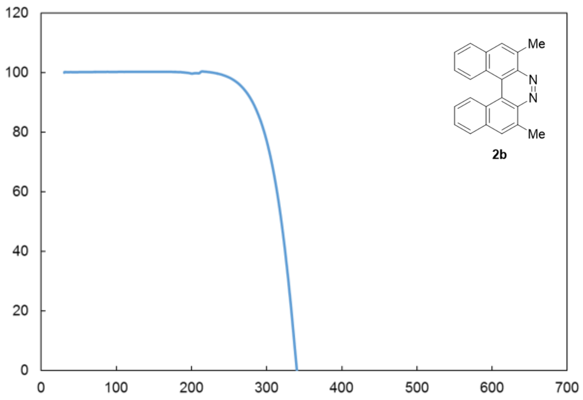

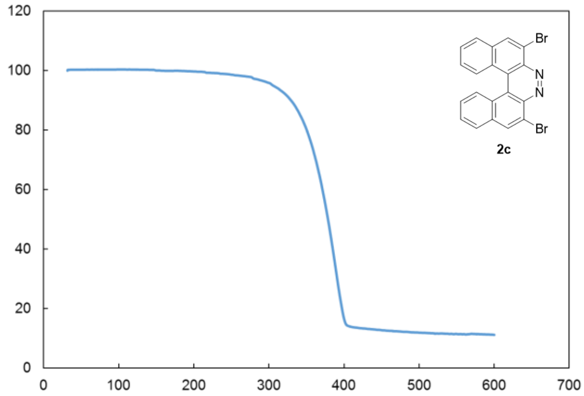

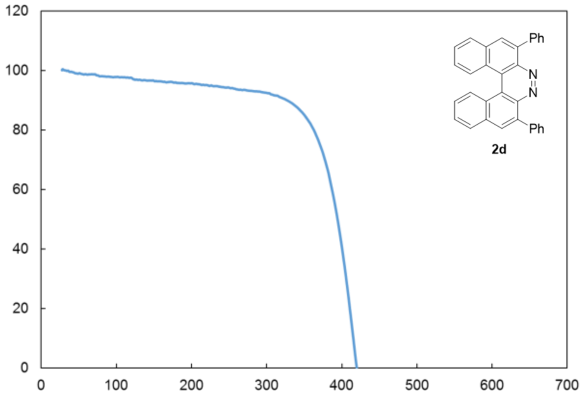

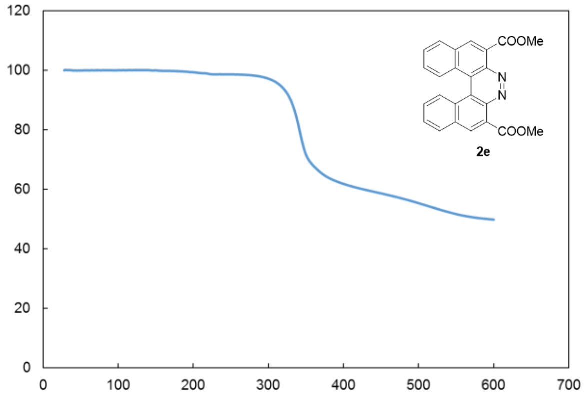
120
100
80
60
40
20
0
0
100
200
300
400
500
600
700
COOMe
N
N
COOMe
2e

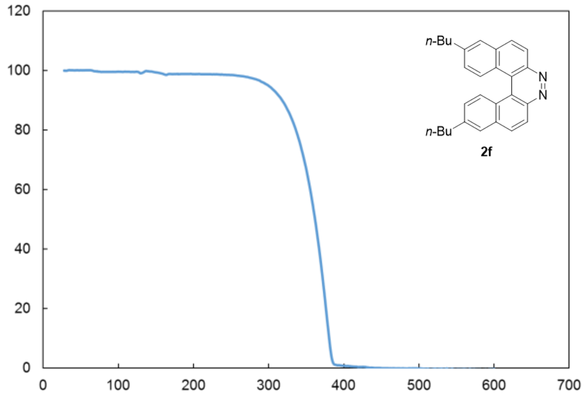
120
100
80
60
40
20
0
0
100
200
300
400
500
600
700
n-Bu
N
N
n-Bu
2f

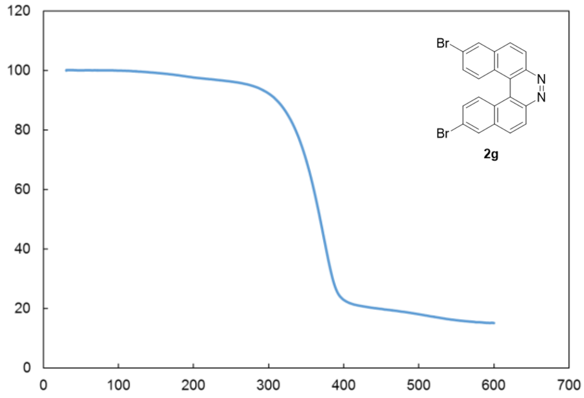
120
100
80
60
40
20
0
0
100
200
300
400
500
600
700
Br
N
N
Br
2g

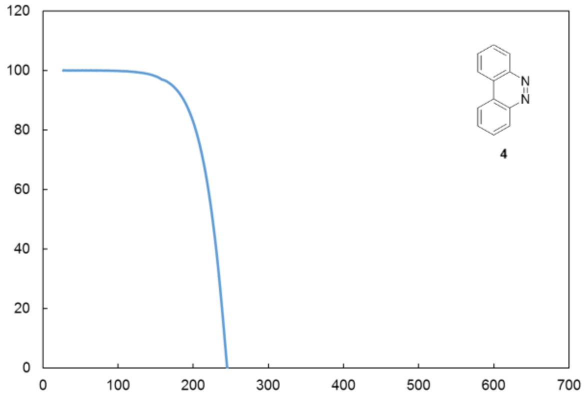
120
100
80
60
40
20
0
0
100
200
300
400
500
600
700
N
N
4

^{1}H and ^{13}C NMR spectra

^{1}H NMR: (400 MHz, $CDCl_3$)

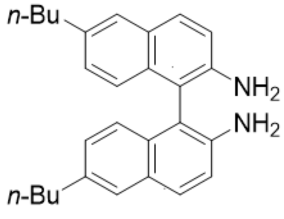

1f

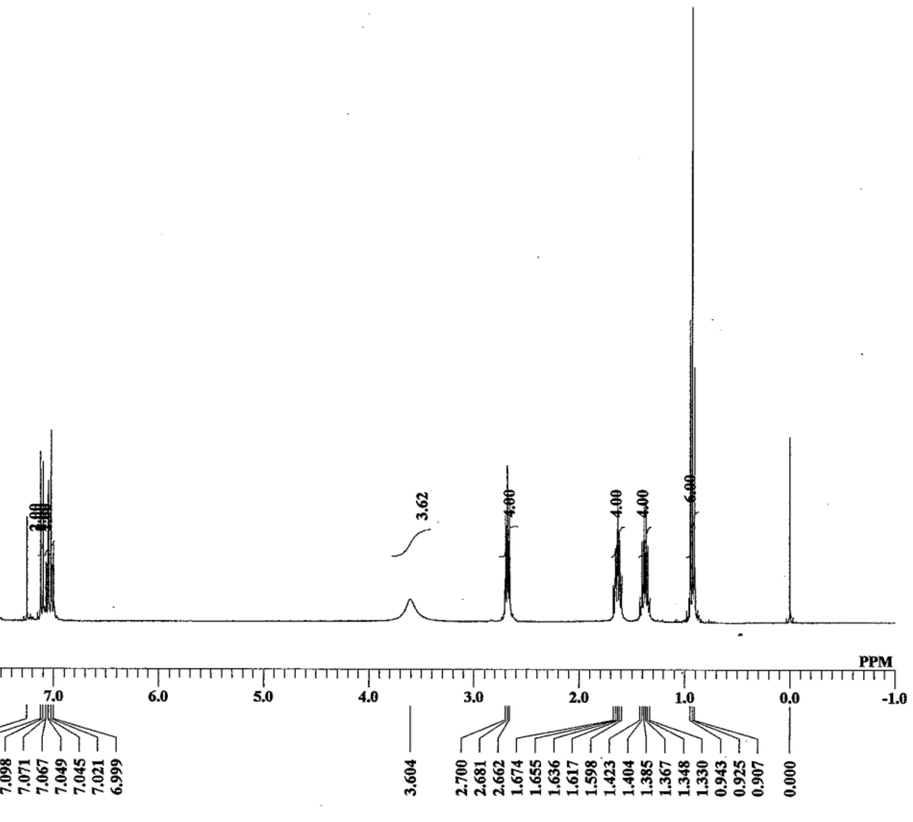

^{13}C NMR: (100 MHz, $CDCl_3$)

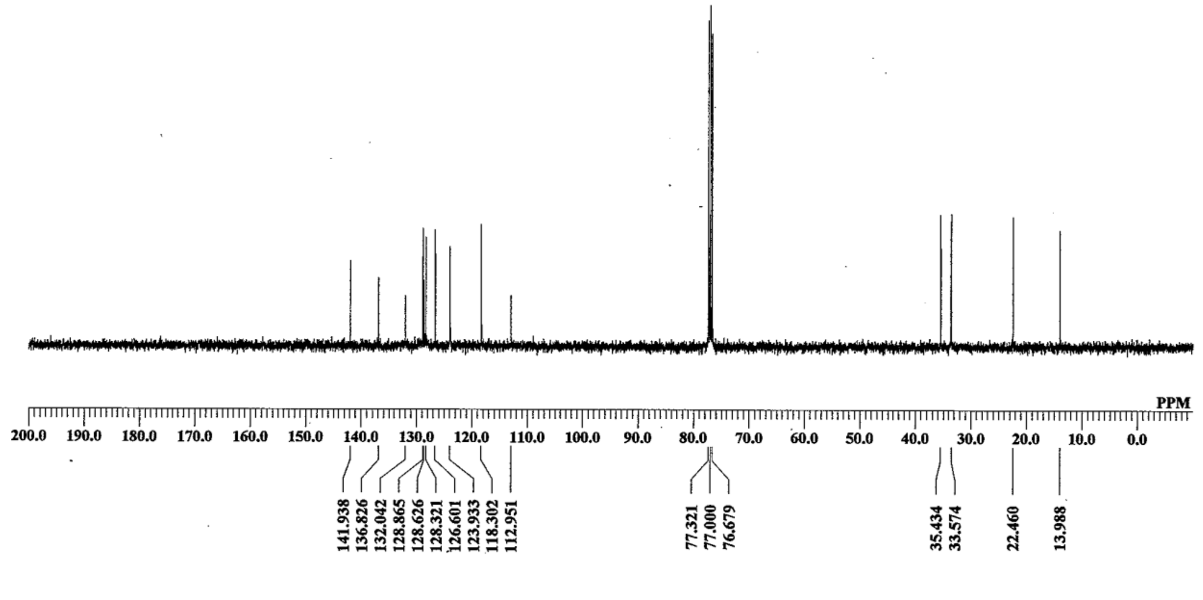

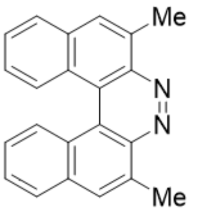

2b

^{1}H NMR: (400 MHz, $CDCl_3$)

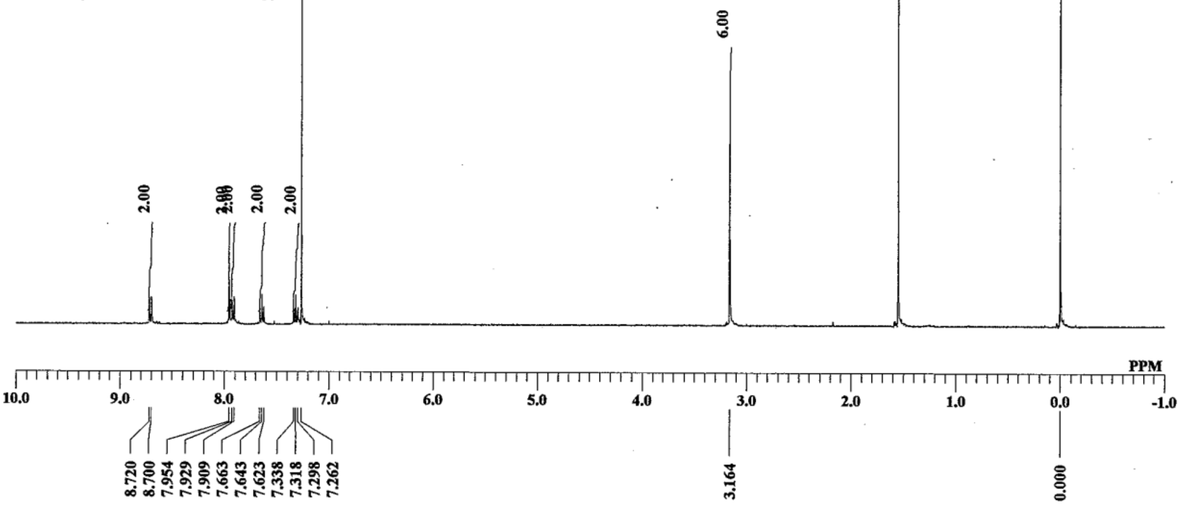

^{13}C NMR: (100 MHz, $CDCl_3$)

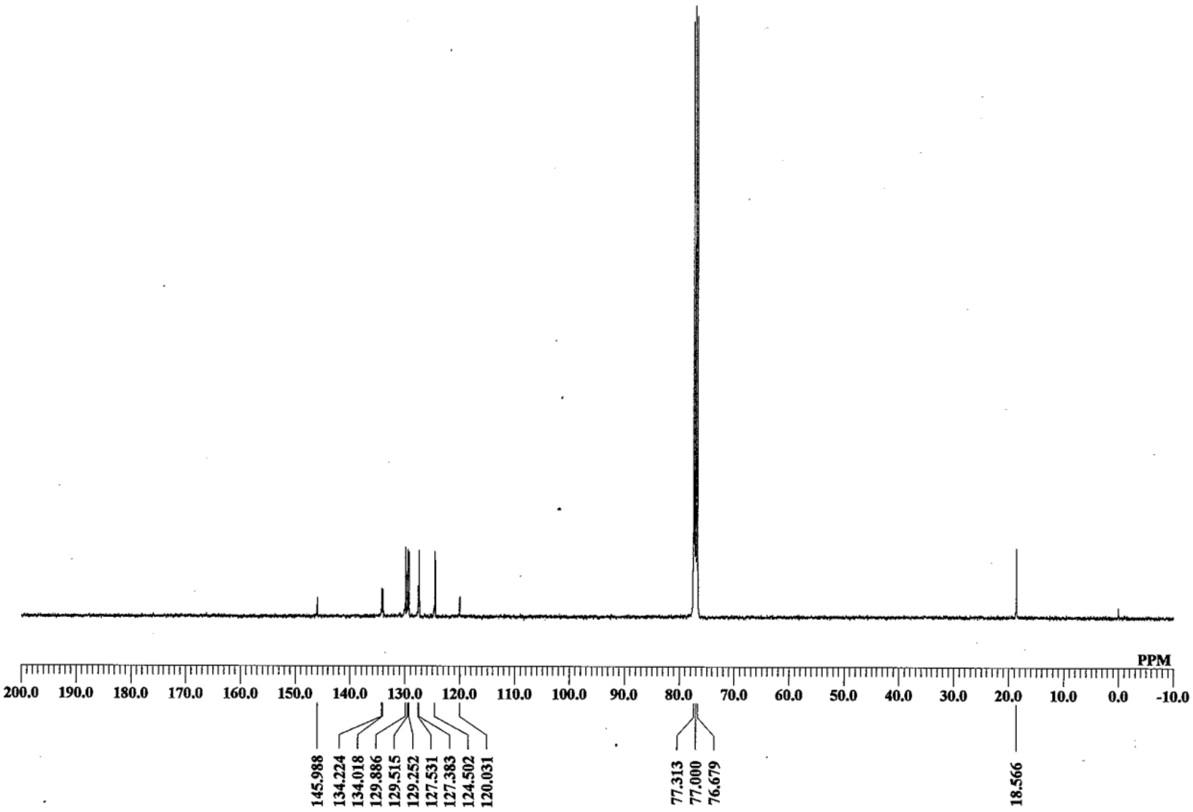

^{1}H NMR: (400 MHz, $CDCl_3$)

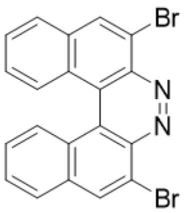

2c

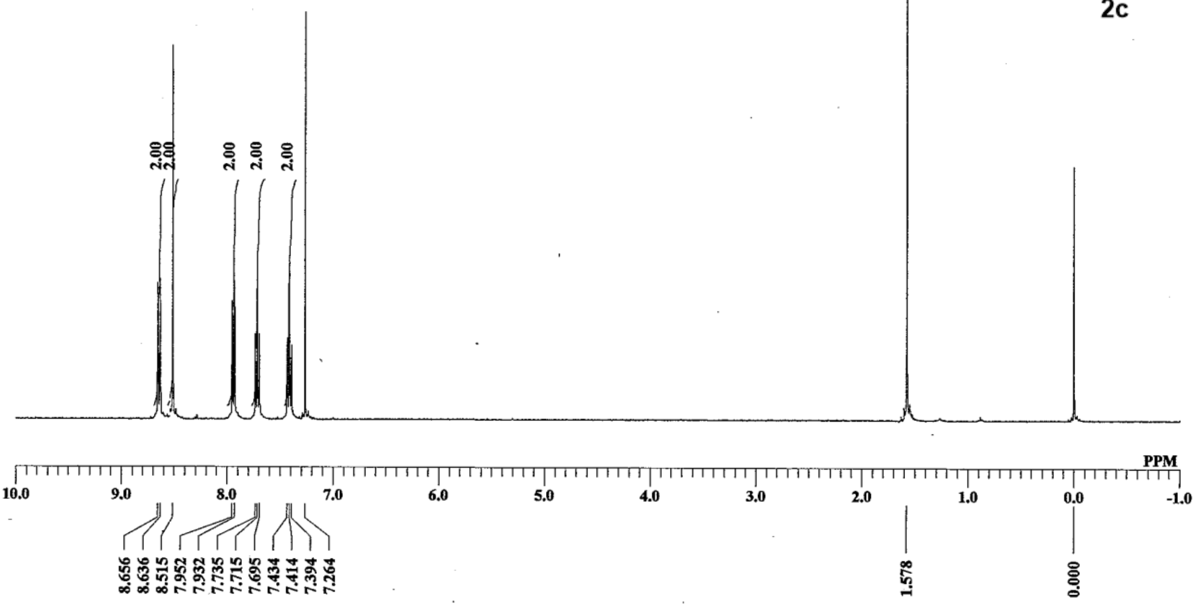

^{13}C NMR: (100 MHz, $CDCl_3$)

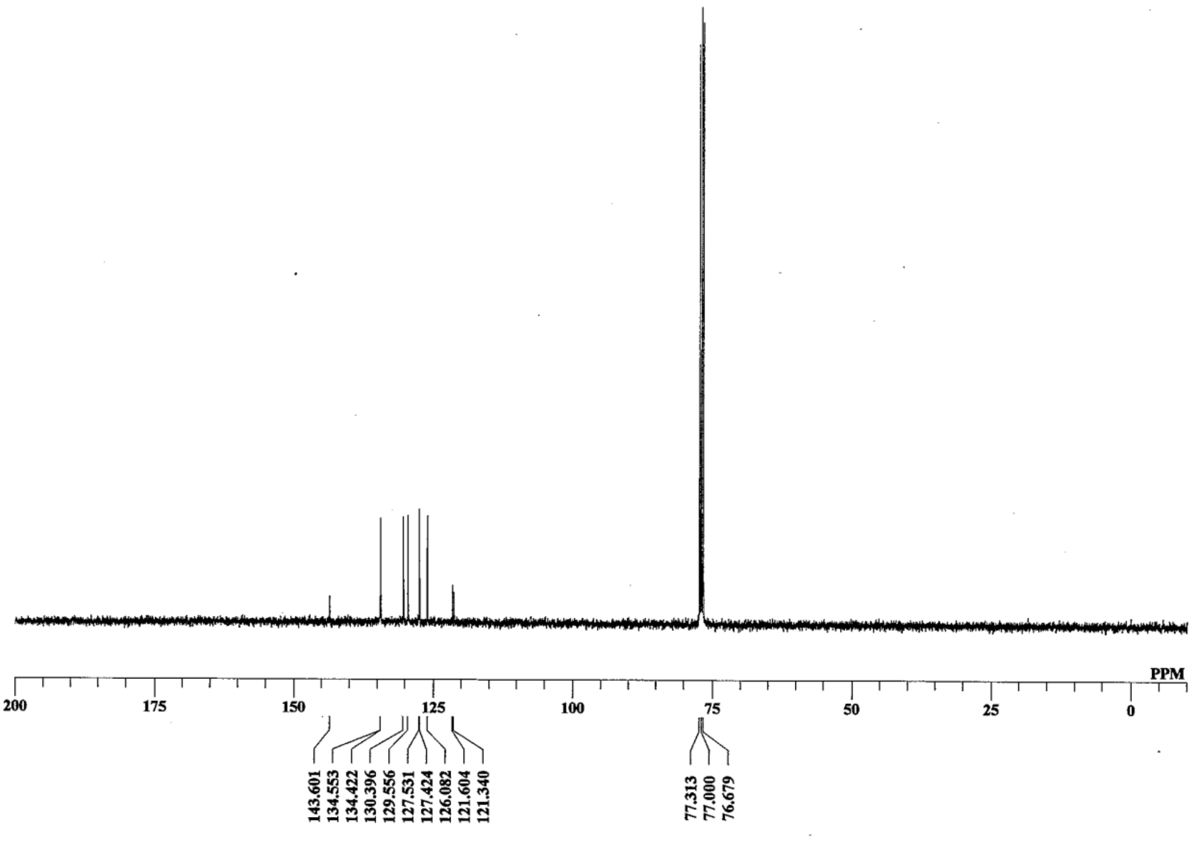

^{1}H NMR: (400 MHz, $CDCl_3$)

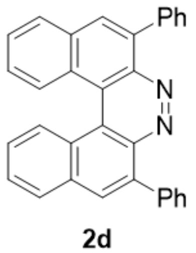

2d

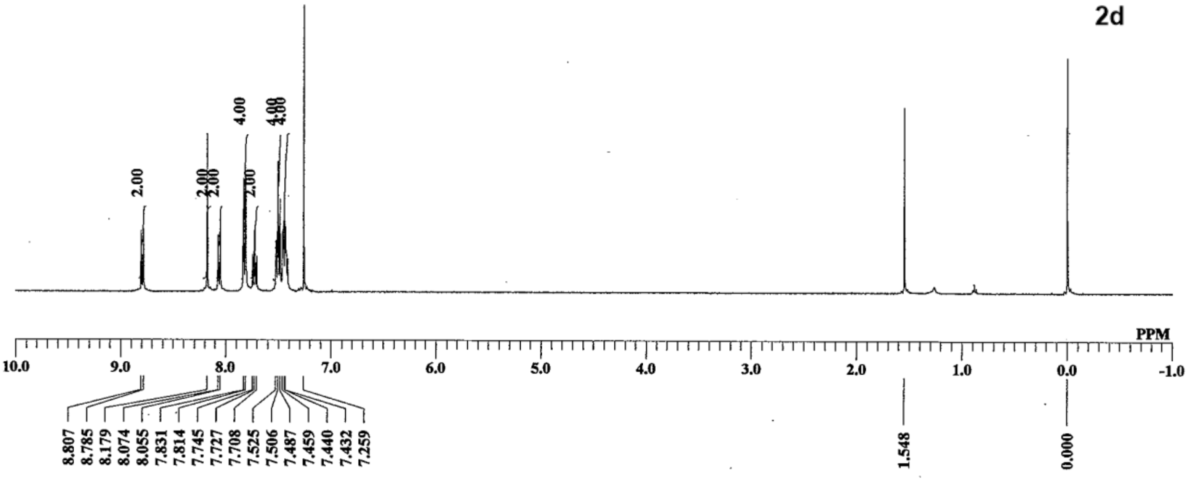

^{13}C NMR: (100 MHz, $CDCl_3$)

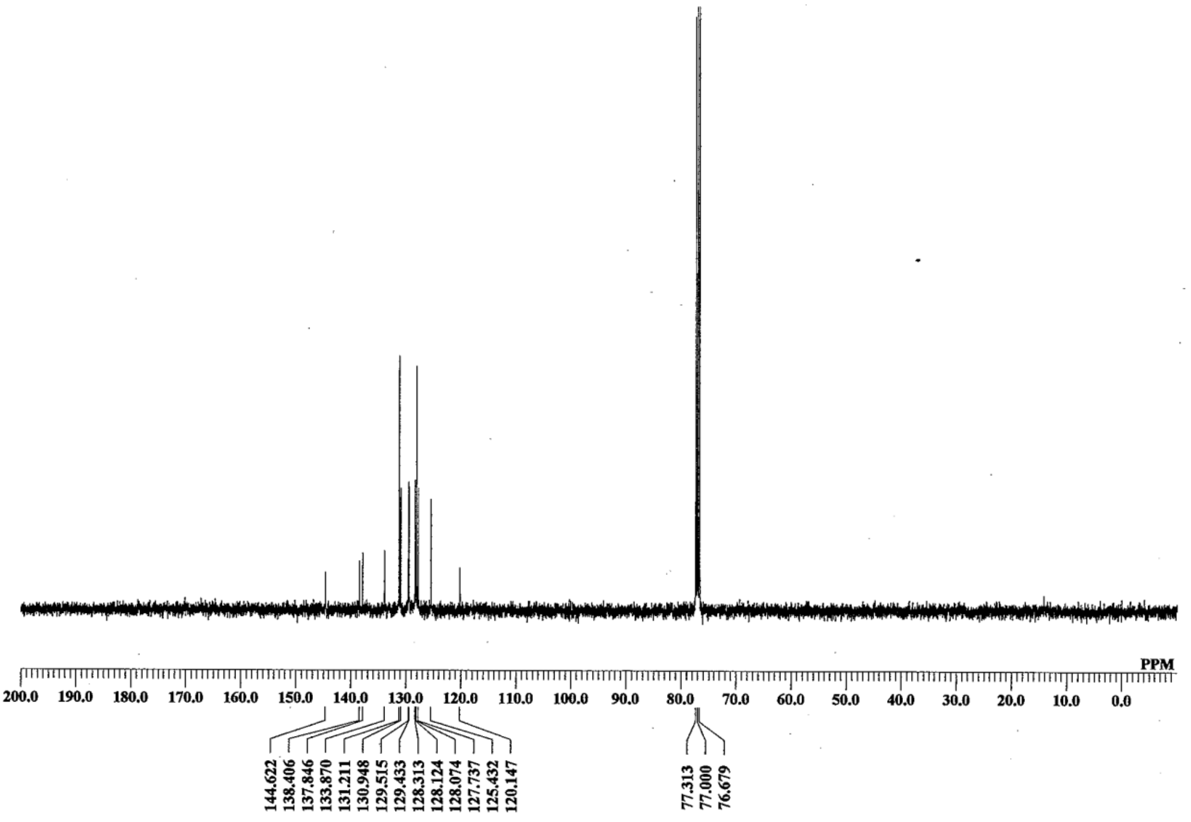

^{1}H NMR: (400 MHz, $CDCl_3$)

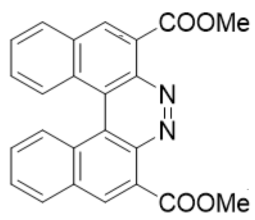

2e

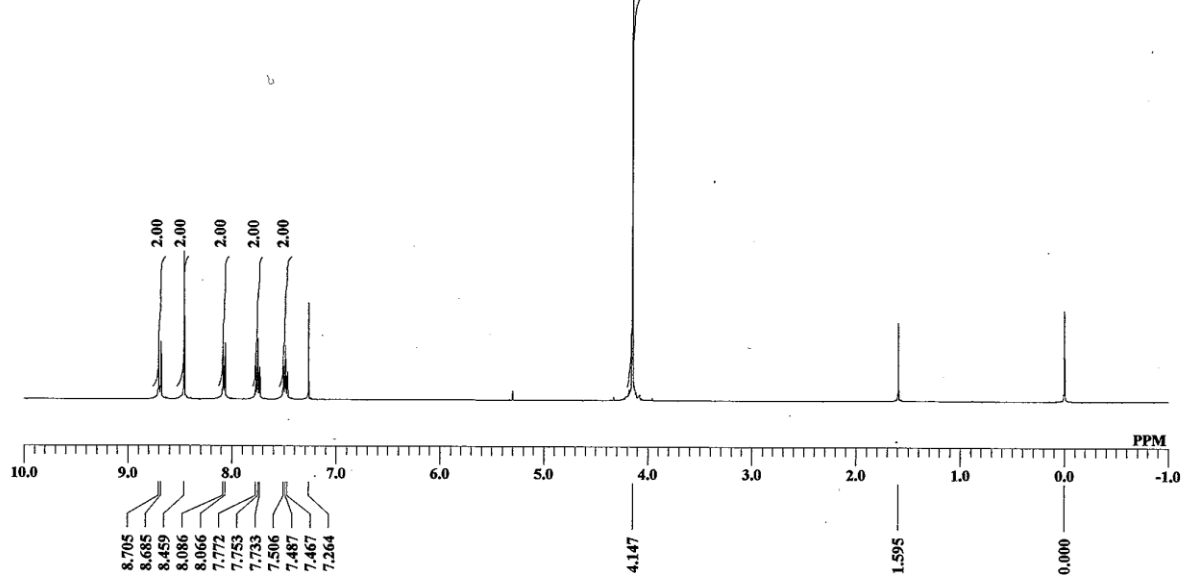

^{13}C NMR: (100 MHz, $CDCl_3$)

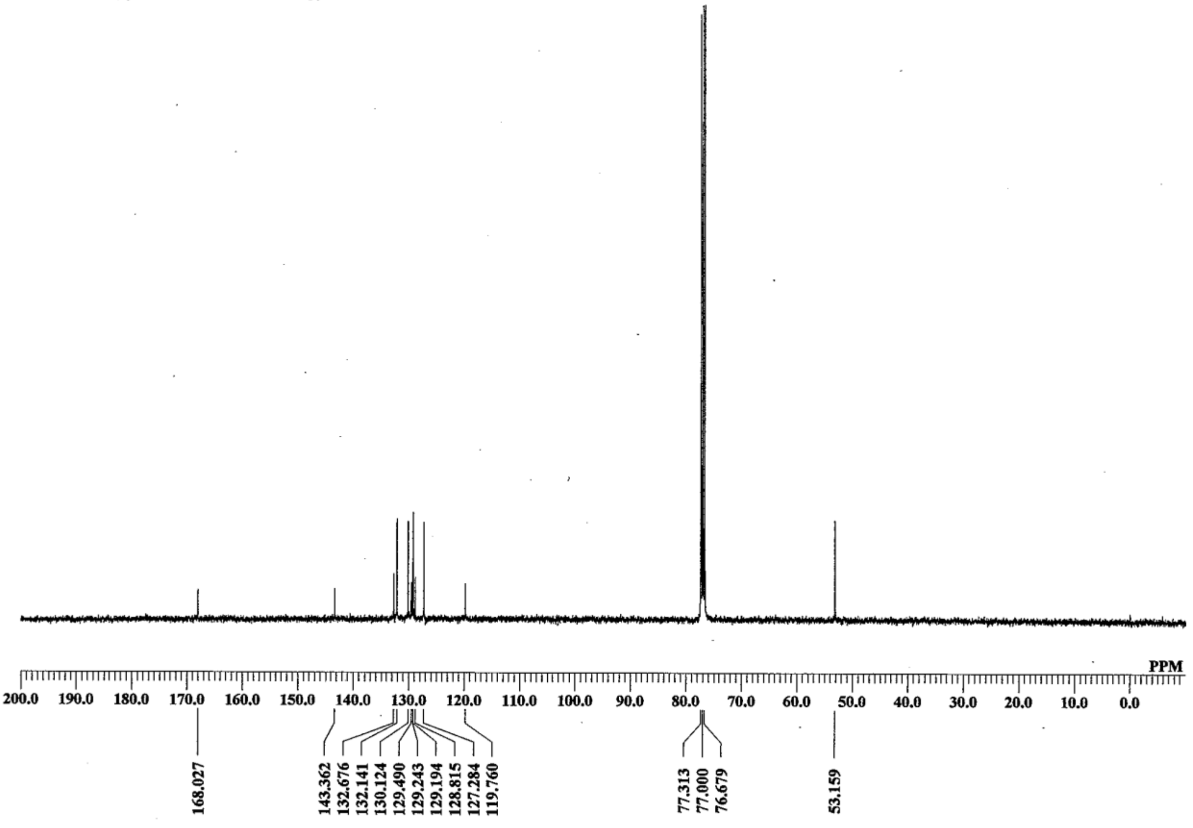

^1H NMR: (400 MHz, $CDCl_3$)

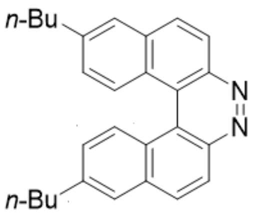

2f

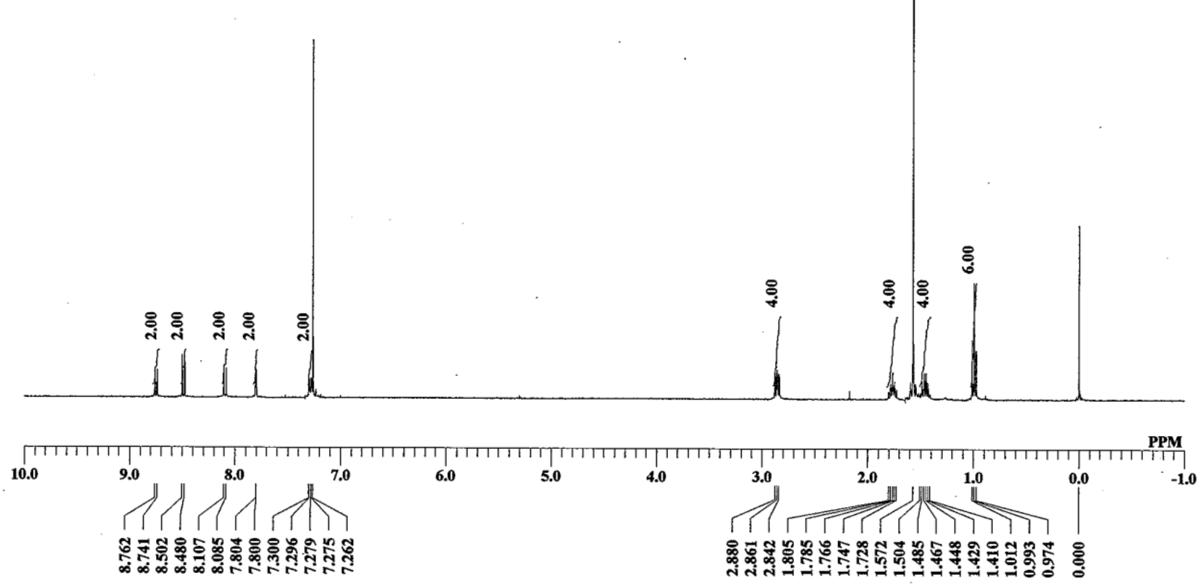

^{13}C NMR: (100 MHz, $CDCl_3$)

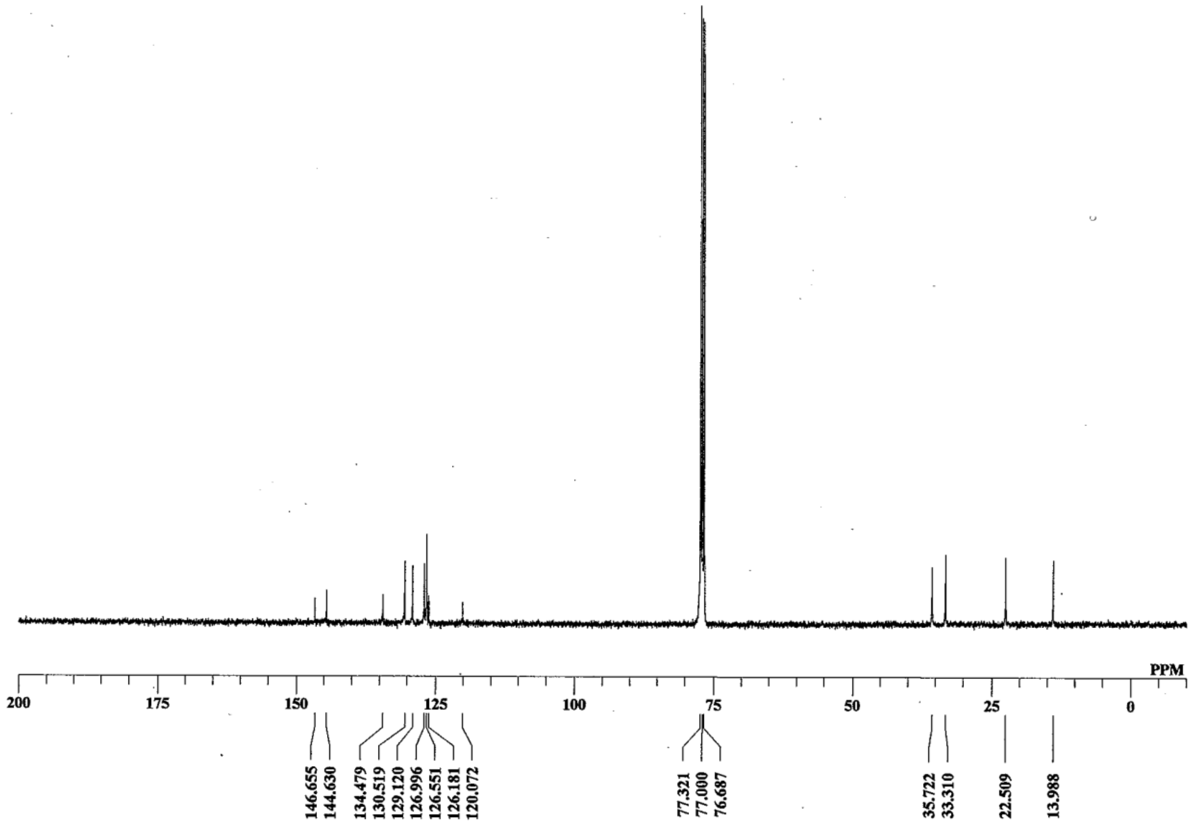

^{1}H NMR: (400 MHz, CDCl$_3$)

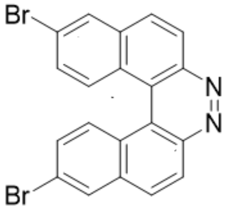

2g

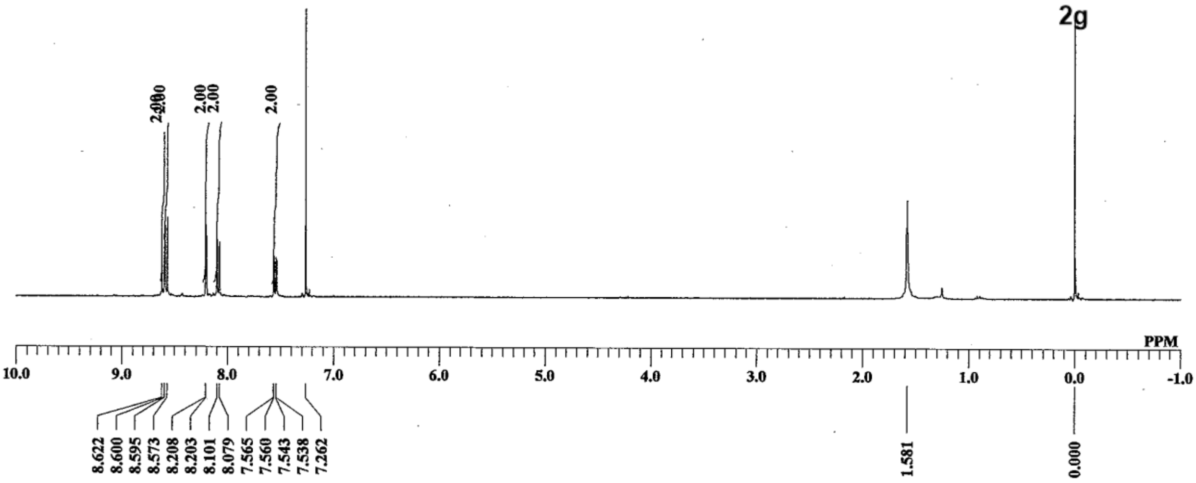

^{13}C NMR: (100 MHz, CDCl$_3$)

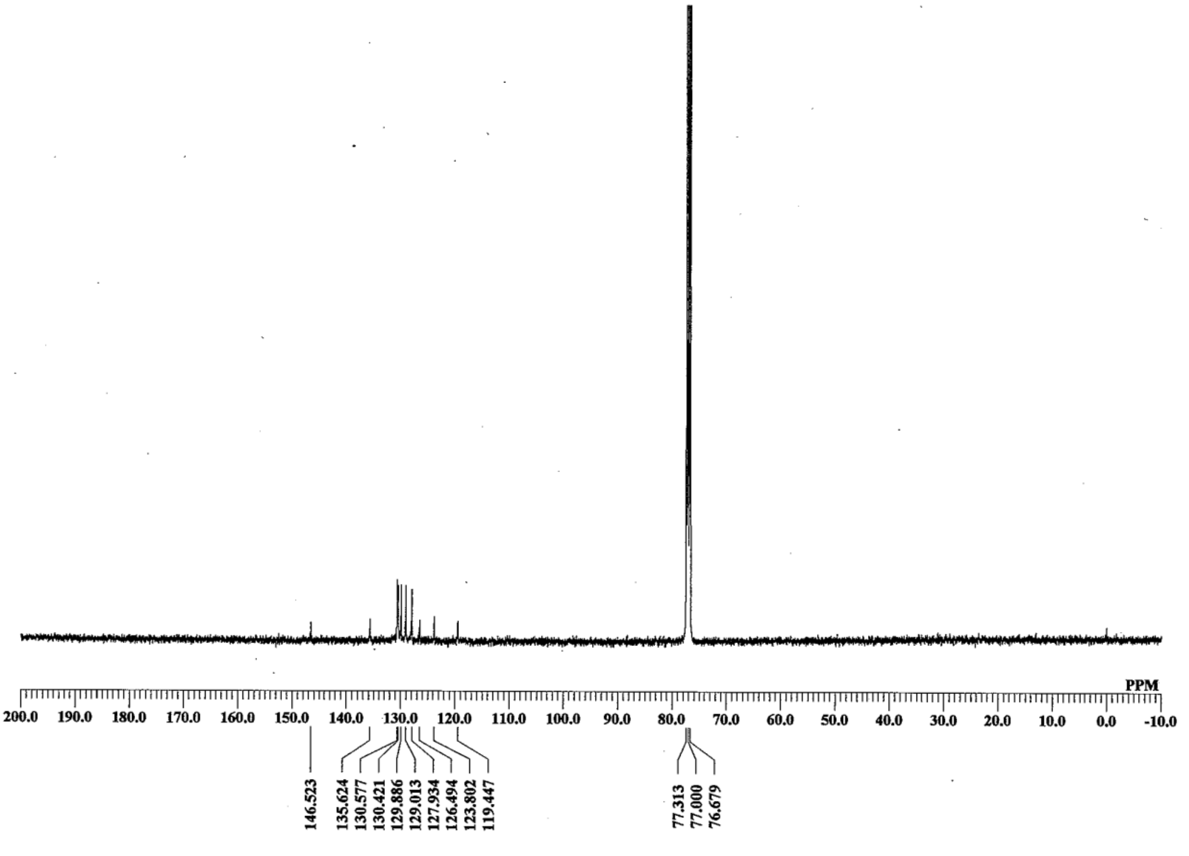

References

S1 Mikami, K.; Korenaga, T.; Yusa, Y.; Yamanaka, M. *Adv. Synth. Catal.* **2003**, *345*, 246.

S2 Kano, T.; Tanaka, Y.; Osawa, K.; Yurino, T.; Maruoka, K. *J. Org. Chem.* **2008**, *73*, 7387.

S3 Scarborough, C. C.; McDonald, R. I.; Hartmann, C.; Sazama, G. T.; Bergant, A.; Stahl, S. S. *J. Org. Chem.* **2009**, *74*, 2613.

S4 (a) Taffarel, E.; Chirayil, S.; Thummel, R. P. *J. Org. Chem.* **1994**, *59*, 823. (b) Smrcina, M.; Vyskocil, S.; Maca, B.; Polasek, M.; Claxton, T. A.; Abbott, A. P.; Kocovsky, P. *J. Org. Chem.* **1994**, *59*, 2156.

S5 (a) Vilches-Herrera, M.; Miranda-Sepúlveda, J.; Rebolledo-Fuentes, M.; Fierro, A.; Lühr, S.; Iturriaga-Vasquez, P.; Cassels, B. K.; Reyes-Parada, M. *Bioorg. Med. Chem.* **2009**, *17*, 2452. (b) Yan, P.; Millard, A. C.; Wei, M.; Loew, L. M. *J. Am. Chem. Soc.* **2006**, *128*, 11030.

S6 Dehghanpour, S.; Afshariazar, F.; Assoud, J. *Polyhedron* **2012**, *35*, 69.

S7 Manolikakes, G.; Hernandez, C. M.; Schade, M. A.; Metzger, A.; Knochel, P. *J. Org. Chem.* **2008**, *73*, 8422.

S8 Caronna, T.; Fontana, F.; Mele, A.; Sora, I. N.; Panzeri, W.; Viganò, L. *Synthesis* **2008**, 413.

S9 Takeda, Y.; Okazaki, M.; Minakata, S. *Chem. Commun.* **2014**, *50*, 10291.

S10 Holt, P. F.; Smith, A. E. *J. Chem. Soc.* **1965**, 7088.

S11 Bjørsvik, H. R.; González, R. R.; Liguori, L. *J. Org. Chem.* **2004**, *69,* 7720.
